# A phylogenetic study to assess the link between biome specialization and diversification in swallowtail butterflies

**DOI:** 10.1111/gcb.16344

**Published:** 2022-07-23

**Authors:** Sara Gamboa, Fabien L. Condamine, Juan L. Cantalapiedra, Sara Varela, Jonathan S. Pelegrín, Iris Menéndez, Fernando Blanco, Manuel Hernández Fernández

**Affiliations:** ^1^ Centro de Investigación Mariña (CIM) Universidade de Vigo, Grupo de Ecoloxía Animal (GEA), MAPAS Lab Vigo Pontevedra Spain; ^2^ Departamento de Geodinámica, Estratigrafía y Paleontología, Facultad de Ciencias Geológicas Universidad Complutense de Madrid Madrid Spain; ^3^ Departamento de Cambio Medioambiental Instituto de Geociencias (UCM, CSIC) Madrid Spain; ^4^ CNRS UMR 5554 Institut des Sciences de l'Evolution de Montpellier (Université de Montpellier) Montpellier France; ^5^ Departamento de Ciencias de la Vida, Edificio de Ciencias Campus Científico‐Tecnológico Universidad de Alcalá Alcalá de Henares Spain; ^6^ Área de Biología y Ciencias Ambientales Facultades de Ciencias Básicas y Educación Universidad Santiago de Cali Santiago de Cali Valle del Cauca Colombia; ^7^ Departamento de Biología, Facultad de Ciencias Naturales y Exactas Universidad del Valle, Campus Meléndez Santiago de Cali Valle del Cauca Colombia; ^8^ Museum für Naturkunde, Leibniz‐Institut für Evolutions und Biodiversitätsforschung Berlin Germany

**Keywords:** bioclimatology, ecological specialization, macroecology, macroevolution, Papilionidae, resource‐use, speciation

## Abstract

The resource‐use hypothesis, proposed by E.S. Vrba, states that habitat fragmentation caused by climatic oscillations would affect particularly biome specialists (species inhabiting only one biome), which might show higher speciation and extinction rates than biome generalists. If true, lineages would accumulate biome‐specialist species. This effect would be particularly exacerbated for biomes located at the periphery of the global climatic conditions, namely, biomes that have high/low precipitation and high/low temperature such as rainforest (warm‐humid), desert (warm‐dry), steppe (cold‐dry) and tundra (cold‐humid). Here, we test these hypotheses in swallowtail butterflies, a clade with more than 570 species, covering all the continents but Antarctica, and all climatic conditions. Swallowtail butterflies are among the most studied insects, and they are a model group for evolutionary biology and ecology studies. Continental macroecological rules are normally tested using vertebrates, this means that there are fewer examples exploring terrestrial invertebrate patterns at global scale. Here, we compiled a large Geographic Information System database on swallowtail butterflies' distribution maps and used the most complete time‐calibrated phylogeny to quantify diversification rates (DRs). In this paper, we aim to answer the following questions: (1) Are there more biome‐specialist swallowtail butterflies than biome generalists? (2) Is DR related to biome specialization? (3) If so, do swallowtail butterflies inhabiting extreme biomes show higher DRs? (4) What is the effect of species distribution area? Our results showed that swallowtail family presents a great number of biome specialists which showed substantially higher DRs compared to generalists. We also found that biome specialists are unevenly distributed across biomes. Overall, our results are consistent with the resource‐use hypothesis, species climatic niche and biome fragmentation as key factors promoting isolation.

## Graphical abstract

After the resource‐use hypothesis, habitat fragmentation caused by climatic changes would affect particularly species inhabiting only one biome, which might suffer more both speciation and extinction events. This effect would be more substantial for biomes with high/low precipitation and temperature. We tested these hypotheses in swallowtail butterflies. Continental macroecological rules are normally tested using vertebrates, so there are fewer examples studying invertebrates. We found that swallowtails present a greater number of biome specialists than biome generalists and they showed higher diversification rates. Also, extreme biomes showed more specialist species and tropical‐woodland, steppe and tundra specialists showed the highest diversification values.

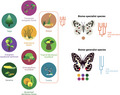

## 1 INTRODUCTION

Geographical patterns of species distribution are determined by current climatic conditions and by long‐term historical and macroevolutionary processes (Barnosky, 2001; Lomolino et al., 2016; Wiens & Donoghue, 2004). Climate shifts influence the expansion and contraction of biomes and the subsequent establishment or demise of ecological and geographic barriers, conditioning the evolution of life forms worldwide (Scheffers et al., 2016; Tian et al., 2018). Some theories point to these large‐scale processes as major forces triggering faunal turnover, in contrast to biotic interactions (Benton, 2009). In this regard, the resource‐use hypothesis (Vrba, 1980, 1987) highlights the role of biomes, and their fragmentation–expansion dynamics responding to global climatic changes, as arenas for macroevolutionary processes (i.e. speciation and extinction; see Hernández Fernández & Vrba, 2005). According to this hypothesis, large‐scale environmental changes result in biome fragmentation and promote diversification, particularly among biome‐specialist species (Maguire & Stigall, 2008). On the contrary, biome‐generalist species might be less impacted by global changes.

Furthermore, the resource‐use hypothesis predicts that biomes at the extremes of the climatic gradient should be more affected by global climatic changes and the associated fragmentation events (Hernández Fernández & Vrba, 2005; Vrba, 1992). If this is true, there should be an overrepresentation of biome‐specialist species as a result of vicariance and speciation processes (Hernández Fernández & Vrba, 2005). In this context, extreme biomes are the ones located at the extreme of the variation of the two major characterizing climatic variables, temperature and aridity. These include the ones that are traditionally considered as ‘harsh’ such as subtropical desert (warm‐arid), steppe (cold‐arid) and tundra (cold‐humid) but also the evergreen equatorial rainforest, which is a warm biome located at the extreme of the precipitation variable (Hernández Fernández & Vrba, 2005). The rainforest, although it is usually considered as a very stable climate across time (Pennington et al., 2015; Whitmore, 1998), represents the warm‐humid extreme of the Earth's climates and is greatly affected by climatic fluctuations and global aridity phases, which can lead to fragmentation of these forests (Brée et al., 2020; Onstein et al., 2018; Whitmore, 1998; Wüster et al., 2005).

When considering a possible relationship between species biome specialization and diversification rates (DRs), it is important to consider the potential effect of species distribution areas. A relationship between the number of biomes occupied by a species and its distribution area is expected. The broader the range of a species, the more likely its range includes different biomes, while species with a narrower distribution are more likely to evolve as endemic of a single biome. Furthermore, several authors proposed a direct correlation between species age and area, where species would have increasing sizes of their distribution ranges the longer they persisted (Willis, 1922). In general terms, this idea has been rejected, as there are numerous examples of widely distributed young species and old species with very restricted distributions (Gaston, 2003), and most authors consider that range sizes expand relatively rapidly after speciation to, perhaps more gradually, decline as species age (Webb & Gaston, 2000). Nevertheless, other works have proposed that there is a positive correlation between species area and age, and therefore DR, at least among closely related species or genera (Miller, 1997; Taylor & Gotelli, 1994).

The resource‐use hypothesis has been previously tested in different vertebrate groups showing a high prevalence of biome specialists (Cantalapiedra et al., 2011; Gómez Cano et al., 2013; Hernández Fernández et al., 2022; Hernández Fernández & Vrba, 2005; Moreno Bofarull et al., 2008; Vrba, 1987) and a relationship between bioclimatic specialization and higher speciation rates (Cantalapiedra et al., 2011; Menéndez et al., 2021). However, the large‐scale biogeographic patterns of non‐vertebrate groups are poorly explored and barely used as models to test macroecological rules.

The globally distributed swallowtail butterflies (family Papilionidae) constitute a species‐rich group including 32 genera and more than 570 described species (Scriber et al., 1995; Tyler et al., 1994). Although most species are found in tropical regions, the ecological diversity of Papilionidae also includes some lineages adapted to temperate and even cold environments (Condamine et al., 2012; Condamine, Nabholz, et al., 2018). Moreover, as one of the best known and broadly studied insect groups, swallowtails are recognized as model organisms in evolutionary biology, ecology, genetics and conservation biology (e.g. Collins & Morris, 1985; Condamine et al., 2012; Scriber et al., 1995). As statistical models and phylogenetic data become more available for studying evolutionary processes (Allio et al., 2021), they can be used to test the relationship between DRs and climatic variables across their geographical range (e.g. Gómez‐Rodríguez et al., 2015; Kozak & Wiens, 2010).

Here, we tested several predictions of the resource‐use hypothesis in swallowtail butterflies using a phylogenetic approach with the aim to test the universality of the global patterns observed in vertebrates. Swallowtail butterflies, as ectothermic and strictly herbivorous organisms, are expected to directly respond to global climate changes (Bale et al., 2002; Clusella‐Trullas et al., 2011; Kingsolver et al., 2013). Thus, following Vrba's hypothesis, we expected to observe a high proportion of biome specialists. This proportion should reflect underlying higher mean DRs in biome specialists than in biome generalists. We also expected a higher proportion of biome‐specialist species in the biomes representing the extremes of the climatic gradient (rainforest, desert, steppe and tundra) explained by a hypothesized higher degree of contraction and fragmentation processes during the climatic fluctuations of the late Cenozoic. Specialist lineages associated with these biomes should also present higher mean DRs than specialists from other biomes. Finally, we will also test the relationship between species distribution area and DR to consider the effect of area as a possible alternative to the within‐biome specialization expected under the resource‐use hypothesis.

## 2 MATERIALS AND METHODS

### 2.1 Biome, area and phylogenetic data

We worked at a global scale and focused on the butterfly family Papilionidae (Lepidoptera: Papilionoidea), a well‐known family with a worldwide distribution and occurring in most terrestrial environments (Condamine et al., 2012, 2013). We used the most complete phylogeny of Papilionidae to date (Allio et al., 2020, 2021), and considered the species included in all Papilionidae genera and subgenera following several studies (see Appendix S1 in Supporting Information). We focused our study on the current geographical distribution of species. Species distribution ranges, gathered from an extensive review of specialized literature from multiple sources containing distribution maps and information at several scales (see Appendix S2 in Supporting Information), were digitized, georeferenced and summarized using the Geographic Information System software ArcGIS (ESRI, 2018) and QGIS (QGIS Development Team, 2018). In those cases with several sources containing information for the same species, we took in consideration the most updated one. Species distribution areas were quantified using the R software version 4.1.0 (R Core Team, 2021) and the packages *raster* (v3.4‐13; Hijmans, 2021); *rgdal* (v1.5‐25; Bivand et al., 2021) and *maptools* (v1.1‐1; Bivand & Lewin‐Koh, 2021) under the equal‐area Eckert IV projection.

### 2.2 Bioclimatic characterization of the species

**FIGURE 1:**
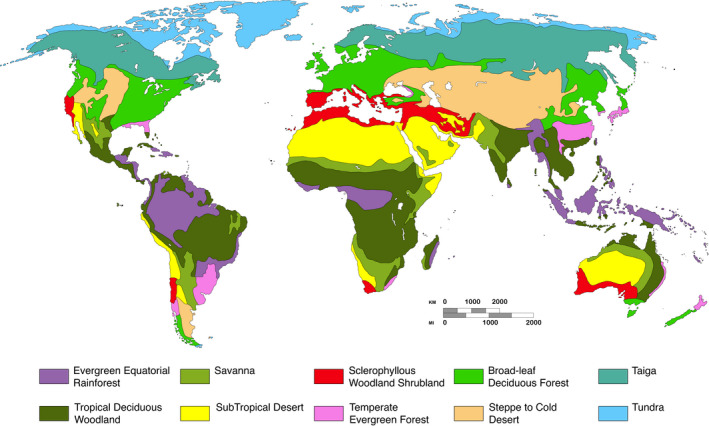
World biomes distribution considered in this work (modified from Walter, 1970).

The biomes inhabited by a species were determined by the overlap between the reported geographical distribution of each species and the biome map (Hernández Fernández, 2001). Here, we consider a biome as inhabited by a species if it constitutes 15% or more of its geographical range. For the cases where the species overlapped isolated, small and distinct biome patches, we also recorded the presence of a species in a biome if the species is present in 50% or more of that biome patch (Hernández Fernández, 2001). Furthermore, for those species with presence in mountain environments, following Moreno Bofarull et al. (2008) and Cantalapiedra et al. (2011), we considered the altitudinal vegetation belts (ETOPO2v2, NOAA National Geophysical Data Center, 2006), which were not included in Walter's map (Figure 1). The overlap between species distribution ranges and biomes was calculated using ArcGIS software. These criteria allow to represent the adaptation capacity of species while maintaining their climatic specificity and, at the same time, allow us to compare our results with previous works using the same methodology (Cantalapiedra et al., 2011; Gómez Cano et al., 2013; Hernández Fernández et al., 2022; Hernández Fernández & Vrba, 2005; Menéndez et al., 2021; Moreno Bofarull et al., 2008). Then, we computed the biomic specialization index (BSI) defined by Hernández Fernández and Vrba (2005) as the number of inhabited biomes by a species. Biome‐specialist species were defined as those occupying only one biome, with a BSI = 1 (Hernández Fernández & Vrba, 2005). In turn, species with BSI >1 were considered as biome generalists, differentiating between ‘semi‐generalists’ (1 < BSI < 5) and ‘extreme generalists’ (BSI ≥5) (Hernández Fernández & Vrba, 2005).

### 2.3 Biome specialization

We tested the resource‐use hypothesis, which predicts the uneven distribution of biome specialists and biome generalists across biomes, against null models where biome specialization was randomly distributed. In addition to the analysis of the whole family, we partitioned the data according to the recognized subfamilies (Condamine et al., 2012; Nazari et al., 2007). The monotypic subfamily Baroniinae was not considered because it is composed of the single Mexican species *Baronia brevicornis* (inhabitant of tropical deciduous woodlands), which was not enough for statistical purposes.

We compared the observed proportion of biome‐specialist and biome‐generalist species with null models generated by 10,000 Monte Carlo randomizations of the observed data (Gotelli, 2000). These data were coded as a binary matrix representing the presence or absence of every species in each biome. Since specific ecological features of each biome affect species richness (Jetz & Fine, 2012), the randomization we conducted placed species presences in biomes randomly while constraining the overall observed species richness of each biome, which generates changes in the degree of biomic specialization of those species. Finally, simulated samples of BSI incidence were obtained and the significance (*p*‐value) of observed BSI distribution was calculated comparing observed and simulated proportions (Hernández Fernández & Vrba, 2005). All analyses were performed using the R environment (R Core Team, 2021).

### 2.4 Species‐specific DRs and biome specialists

To assess the relationship between diversification and the level of biome occupancy, we estimated species‐specific DRs, or ‘tip rates’. Tip rates are widely used to study DR variation in relation to geography, ecology and phenotypes (Title & Rabosky, 2019). We calculated tip rates from a swallowtail phylogeny (Allio et al., 2021), which includes 391 swallowtail species (~70%), representing all genera and subgenera including the only Baroniinae species *B. brevicornis*, 75 out of 76 Parnassiinae species, and 315 out of ~515 Papilioninae species.

To estimate species‐specific DRs, we used species‐level lineage DRs (Jetz et al., 2012) using the *evol.distinct* function in the R package *picante* (v1.8.1; Kembel et al., 2010). DR is a summary statistic that infers speciation rates for all tips in the phylogeny without requiring a formal parametric inference model and is based on the mean equal splits measure of evolutionary isolation (Redding & Mooers, 2006). DR values were estimated according to the number of splitting events and the internode distances of those branches going from each tip to the tree's root, giving greater weight to branches and splitting events closer to the present (Jetz et al., 2012). When applied to extant species phylogenies (ultrametric phylogenies), DR analyses represent net diversification (i.e. speciation minus extinction; Cantalapiedra et al., 2017).

We assessed the significance of the relationship between species DRs and the number of biomes occupied using phylogenetic generalized least squares (PGLS) to estimate the expected covariance in cross‐species data while controlling for potential phylogenetic signal (Mundry, 2014). PGLS were performed using the R package *caper* (v1.0.1; Orme et al., 2018) and estimating the lambda parameter using the maximum likelihood function. We used phylogenetic analysis of variance (ANOVA) to test the existence of significant differences in biome‐specialist DRs among the different biomes. Phylogenetic ANOVA test was performed using the R packages *phytools* (v0.7‐80; Revell, 2012) and *geiger* (v2.0.7; Pennell et al., 2014).

Moreover, we also explored the relationship between biomic specialization and diversification using the Hidden State Speciation and Extinction (HiSSE) model in the R package *hisse* (v1.9.19; Beaulieu & O'Meara, 2016). In addition to the trait of interest, which differentiated between biome specialists and biome generalists (coded as 1 and 0, respectively), the HiSSE model allowed us to include unobserved factors (hidden states) that could affect diversification. We allowed unlinked rates of speciation (λ0, λ1), extinction (μ0, μ1) and transitions (q01, q10) associated with the two trait states. For the HiSSE model, we set two hidden states (A, B) contained within each observed trait state (i.e. states 0A, 0B, 1A, 1B) so that speciation and extinction rates can vary independently across all four states. Transition rates between all observed and hidden states were also free to vary except for dual transitions (e.g. q0A to q1B, q1A to q0B). We optimized the fit of all models by maximum likelihood and evaluated model performance based on the corrected Akaike information criterion (AICc).

Finally, we explored the impact of species ranges on our results to assess whether a correlation between biome specialization and diversification in swallowtails could be a consequence of the size of the species distribution area. To this effect, we performed a variance partitioning to split the DR variance explained by species distribution area and/or by the number of occupied biomes, using the R package *vegan* (v2.5‐7; Oksanen et al., 2020). Lastly, we tested the significance of the relationships between swallowtail DRs and species distribution areas using a PGLS to consider the potential phylogenetic signal (Mundry, 2014). For the PGLS, the lambda parameter was estimated using the maximum likelihood function.

## 3 RESULTS

### 3.1 Distribution of the BSI

We collected the distribution ranges of a total of 593 swallowtail species from around the world. The group was represented in the 10 terrestrial biomes considered, and in all continents except Antarctica, where there is no evidence of their presence. No swallowtail species inhabits all the 10 biomes, the maximum being the eight biomes occupied by *Papilio polyxenes*.

**FIGURE 2:**
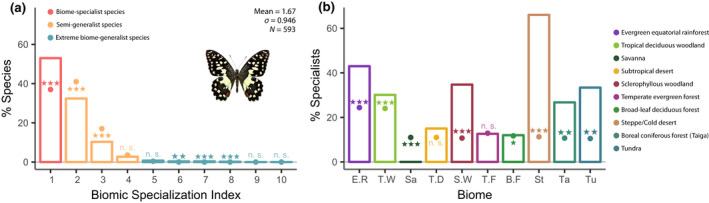
Biomic specialization among Papilionidae: (a) Observed (bars) and simulated (dots) frequency distribution of the biomic specialization index (BSI) in Papilionidae. (b) Observed (bars) and simulated (dots) distribution of biome‐specialist (BSI = 1) Papilionidae species across different biomes. While dots in both figures indicate the expected values by chance, symbols above or below the dots indicate whether observed results (bars) are significantly higher (above) or lower (bellow) than expected by chance with: ***p < .001; **.01 > *p* > .001; *.05 > *p* > .01; n.s. = not significant. *Papilio demoleus* picture by Fabien L. Condamine.

Our results showed that the frequency distribution of BSI was intensely right skewed, with a low mean BSI (BSI = 1.67) (Figure 2a). Overall, 323 species (54.5%) of Papilionidae species inhabit only one biome, and 186 species (31.5%) inhabit two biomes. Moreover, just 1.6% of Papilionidae (nine species) can be considered as extreme biome generalists, inhabiting five or more different biomes (Table S3.1). The American swallowtail (*P. polyxenes*) is the most biome‐generalist species of the group, inhabiting eight different biomes, being only absent in taiga and tundra environments.

Our results showed a significantly high proportion of biome‐specialist species (BSI = 1) (Figure 2a; Table S3.1) while the frequencies of species inhabiting two (BSI = 2) and three (BSI = 3) biomes were significantly lower. The frequencies of species with BSI = 4–5 were not significantly different from those expected under random processes. Nonetheless, the frequencies of species inhabiting six to eight different biomes (BSI = 6–8) were significantly higher than expected.

Both studied subfamilies showed similar distributions of frequencies and proportion of biome specialization to the family species (Figure S3.1; Table S3.2). However, Parnassiinae showed a substantially lower mean BSI (1.36) and a higher proportion of biome‐specialist species (77.6%) than Papilioninae (1.71% and 51.0%, respectively). The frequency of species inhabiting two (BSI = 2) and three (BSI = 3) biomes was significantly low in both subfamilies. Finally, extreme biome‐generalist species were scarce in both subfamilies, with less than 1.6% of Papilioninae and less than 1.4% of Parnassiinae inhabiting five or more different biomes, although the observed values in several of these BSI categories were significantly higher than expected by chance.

### 3.2 Proportion of biome specialists among biomes

Six biomes showed a higher proportion of biome‐specialist species than expected (Figure 2b; Table S3.3). Within the family, 331 papilionid species occur in the equatorial rainforests, of which 43% (143 species) are restricted to it, against 25% expected. In all, 101 biome specialists live exclusively in the tropical deciduous woodland, which constitutes around 30% of all the species present in this biome (25% expected). The sclerophyllous woodland hosts 34.8% of biome specialists, in contrast to 11.1% expected. The steppe is the biome with the highest degree of specialization, harbouring 69% of biome specialists, against 12% expected. Boreal coniferous forest houses six biome‐specialist species, which constitutes around 26% of the total number of species inhabiting this biome, in contrast to 7% expected. On the other hand, two biomes showed a lower proportion of biome‐specialist species than expected: broad‐leaf deciduous forest and savannah, which also constitutes the single biome that hosts no biome‐specialist species.

Papilioninae and Parnassiinae showed substantial differences in their frequencies of biome specialists (Figure S3.2; Table S3.4). Most papilionine biome specialists (about 93% of them) inhabit equatorial rainforest or tropical deciduous woodland, while the vast majority of parnassiine biome specialists are found in the steppe, where biome‐specialist species constitute 88.9% of all the inhabiting parnassians. To a much lesser extent, tropical deciduous woodland and sclerophyllous woodland, with around 50% of biome‐specialist species, also showed a high proportion of biome‐specialist Parnassiinae species. Three biomes host no Papilioninae biome specialists: savannah, steppe and tundra. At the same time, three biomes host no Parnassiinae of any kind: equatorial rainforest, savannah and tropical desert. Moreover, both subfamilies showed a similar percentage of biome specialists in the taiga biome, which was significantly higher than expected.

### 3.3 DRs and biome specialists

**FIGURE 3:**
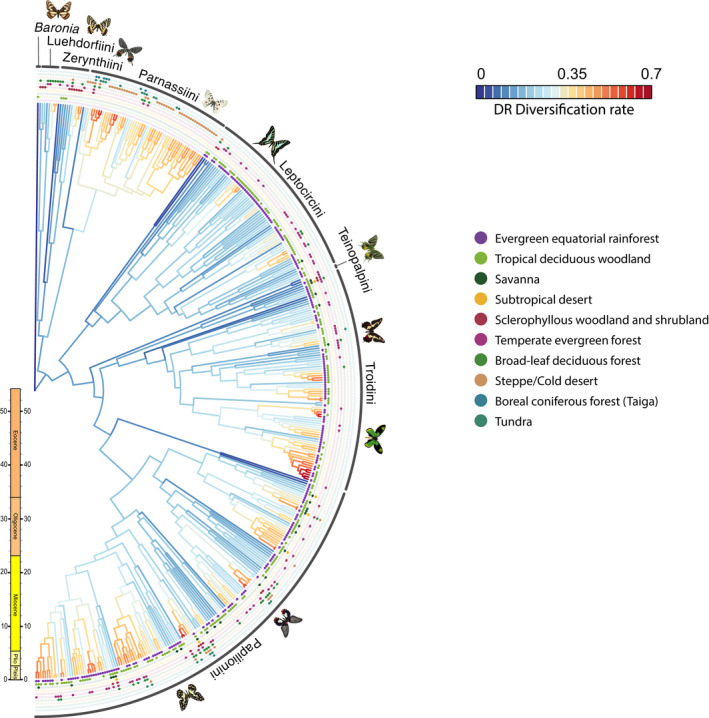
Diversification rate (DR) estimations for Papilionidae as inferred using the DR metric (Jetz et al., 2012). Dots indicate the presence (full) or absence (void) of each species in the considered biomes. Butterfly pictures by Fabien L. Condamine.

The highest diversification values were those from the Papilioninae species *Troides rhadamanthus* and *T. riedeli*, while the lowest value corresponded to *B. brevicornis*, from the monotypic subfamily Baroniinae (Figure 3). Among clades, tribes Leptocircini and Teinopalpini showed the lowest DR values among all Papilioninae, while genus *Parnassius* showed the highest DR values among all Parnassiinae. Regarding the species‐rich genus *Papilio* (tribe Papilionini), the *Pterourus* clade as well as the *memnon* and *aegeus* species groups within the subgenus *Menelaides* also showed high DRs. When separated by subfamilies, Parnassiinae showed higher DR mean values than Papilioninae for every represented BSI.

**FIGURE 4:**
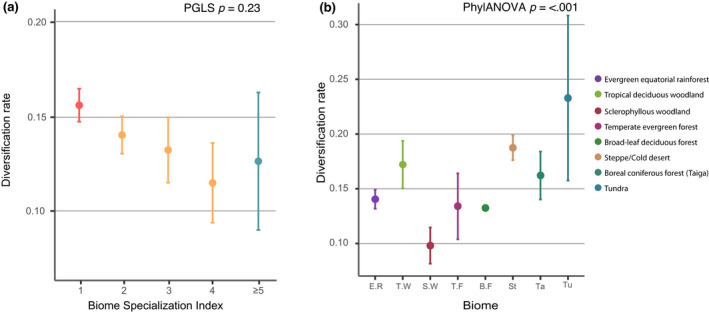
Biome specialists have higher diversification rates (DRs). (a) DRs estimated using the DR metric (Jetz et al., 2012), and grouped by species' biomic specialization index (BSI) for all Papilionidae present in the phylogeny; (b) diversification rate estimated for biome‐specialist species (BSI = 1) present in the phylogeny, divided as a function of the biome they inhabit; Savannah and subtropical desert are not included as there are not specialist species in these biomes. Coloured dots indicate the mean values. Bars correspond to 95% CI. Significance levels (*p*) are provided for phylogenetic generalized least squares and phylogenetic ANOVA analyses of DRs for BSI and biome, respectively. ANOVA, analysis of variance; CI, confidence interval.

Lineage‐specific DR mean values through the different BSI groups (Figure 4; Table S3.5) varied from 0.16 (BSI = 1) to 0.08 (BSI = 6). However, there was a high variability of statistical dispersion among categories, with BSI = 1 and BSI = 2 groups showing generally the smaller standard deviation values than groups of more generalist species.

The high correlation (*R* ^2^ = .86) and the negative slope from PGLS analysis (Table 1) reflected the relationship between occupying a smaller number of biomes and higher rates of diversification among Papilionidae (*p* = .023; Table 1). Biome‐specialist species of different biomes showed significant differences in their DRs (phylANOVA *p* ≤ .001; Table S3.6 in Supporting Information), with tundra specialist species showing the highest DR values, followed by steppe specialists, while sclerophyllous woodland specialists showed the lowest DR values (Figure 4b).

**TABLE 1:** Results of the phylogenetic generalized least squares regressions of species diversification rates and species biome specialization index (BSI) or range area

Variable	Intercept	Slope	Significance	Adjusted *R* ^2^	*λ*
BSI	0.158 ± 0.012	−1.425 ± 0.006	*p* = .023	.810	0.835
Area	0.280 ± 0.036	−0.044 ± 0.001	*p* = .002	.487	0.852

The HiSSE analyses revealed that the model including a hidden effect on diversification of state 1 (biome specialists) was supported against the other SSE models (AICc = 3043.76 vs. AICc = 3058.76 for the second‐best fitting model, ∆AIC = 15.0). Our result showed that biome‐specialist species diversified twice as fast as biome generalists (0.187 vs. 0.099 events/lineage/Myr; Table S3.7 in Supporting Information) and indicated that the diversification of biome specialists was likely influenced by other unmeasured traits.

**FIGURE 5:**
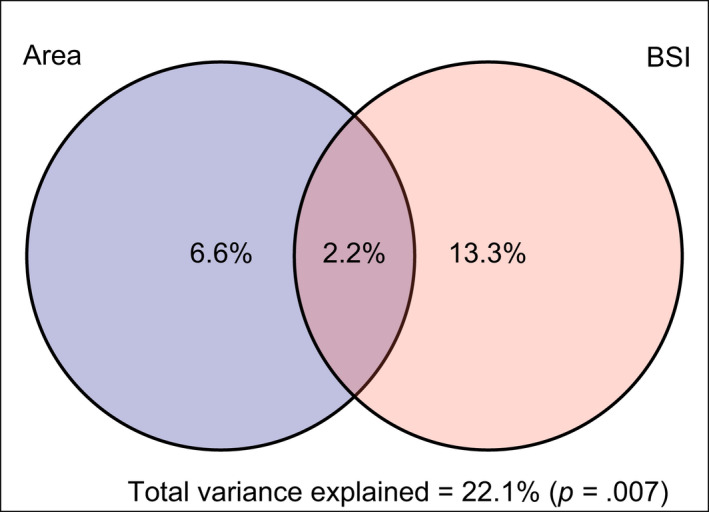
Venn diagram showing the variance partition of conditional and shared effects of species distribution area (left), and species biomic specialization index (right) as fractions of swallowtail diversification rates. Values are percentages of the total variation explained by the full model.

Based on the results of variance partitioning (Figure 5), the effects of BSI (number of biomes occupied by a species) had the most significant unique effect on swallowtail DRs (13.3%), whereas species area explained 6.6% of the total variability. The combined effects of BSI and area variables represented 2.2% of the total variability. The total variance explained by the two variables was 22.1%.

The results of the PGLS showed a negative linear relationship between species area and species DR, meaning that species occupying less area tend to have higher DRs. However, the relationship between species diversification and area, while significant (*p* = .002), had much lower explanatory power than the one showed between species diversification and the number of occupied biomes (BSI; Table 1).

## 4 DISCUSSION

### 4.1 Are there more biome‐specialist swallowtail butterflies than biome generalists?

Globally, our results agree with the first prediction of the resource‐use hypothesis (Vrba, 1980, 1987), showing a high proportion of biome‐specialist species (BSI = 1) (Figure 2a). Apart from biome specialist, we also showed that extreme biome‐generalist species inhabiting more than five biomes were observed infrequently but still more than expected. These results are in line with the results previously obtained by Hernández Fernández and Vrba (2005) for large African mammals, Moreno Bofarull et al. (2008) for South American mammals, Cantalapiedra et al. (2011) for ruminants worldwide, Menéndez et al. (2021) for squirrels of the world and Hernández Fernández et al. (2022) for world's mammals, which suggests that the resource‐use hypothesis provides a common ground for the understanding of evolutionary processes triggered by climatic changes, irrespective of the studied taxa. Moreover, swallowtail butterflies showed a lower mean BSI (they inhabit fewer biomes) than most previously studied mammal groups. Papilionidae butterflies are herbivorous insects with highly specialized host‐plant preferences (Allio et al., 2021; Condamine et al., 2012), and, therefore, their trophic niche could have made them particularly prone to biome specialization (Moreno Bofarull et al., 2008; Vrba, 1980).

### 4.2 Are biome‐specialist swallowtail species evenly distributed across biomes?

Not all biomes showed the same percentage of endemic species. Our results show that some biomes located at the extremes of the climatic conditions, namely tundra, steppe and rainforest have high percentages of biome‐specialist species (more than 33% of their species are endemic; Figure 2b). The rainforest biome stands out by its high number of biome‐specialist species (*n* = 143). The rainforest is an old biome, with more than 60 million years of evolutionary history (Morley, 2011), and with a general pattern of hosting many species for most clades (Condamine et al., 2012; Jaramillo & Cárdenas, 2013; Novotny et al., 2006; Potts & Behrensmeyer, 1992). The steppe and the tundra are relatively modern biome, with less than 15 million years (Abbott & Brochmann, 2003; Barbolini et al., 2020; Friesen et al., 2016) that expanded in the late Neogene‐Quaternary associated with global cooling and orogenic pulses (Horton et al., 2010; Hurka et al., 2019; Strömberg, 2011). The adaptation of the genus *Parnassius* to these biomes, where they are in the majority, should have happened during the late Neogene, meaning that tundra‐ and steppe‐adapted endemic species should be relatively young (Allio et al., 2021; Figure S3.2).

However, our results do not support that the desert biome host many biome‐specialist species (one single endemic species out of seven species inhabits desert environments). This biome, characterized by wide inter‐annual fluctuations in precipitation and by hampering plant–water relationships (Schowalter et al., 1999), constitutes an unfavourable biome for phytophagous insects, like butterflies (Larsen, 1995). In agreement with that idea, a single swallowtail species, *P. saharae*, has specialized in this biome.

Moreover, some non‐extreme biomes also showed high percentages of biome‐specialist species. The prevalence of biome‐specialist species in the tropical deciduous woodlands was pointed out in previous works on mammals (Cantalapiedra et al., 2011; Hernández Fernández et al., 2022; Hernández Fernández & Vrba, 2005; Moreno Bofarull et al., 2008). Even though this biome cannot be considered a climatic extreme, it is a markedly heterogeneous environment whose historical dynamic is closely associated with rainforest fluctuations (Dexter et al., 2018; Haffer, 2008; Hoorn et al., 2010). These dynamics could have provided patches and refugia that promote speciation (Vrba, 1992). In this regard, there are several lineages among Papilionidae characterized by the presence of closely related specialists in the equatorial forests and the tropical deciduous woodlands (Figure 3). Sclerophyllous woodlands and shrublands showed a high proportion of biome specialists related to the high number of species from the Parnassiinae subfamily in this biome (Figure S3.2; Table S3.4), which could be related to the role of the Mediterranean peninsulas as Pleistocene refuges for isolated populations (Dapporto, 2010; Zinetti et al., 2013). Finally, the taiga also showed more specialists than expected (Figure 2b). In this case, taiga specialist swallowtails are linked to mountain systems, which are usually recognized as biodiversity hotspots (Menéndez et al., 2021; Rahbek & Graves, 2001). With these results, we do not find a consistent pattern supporting that, only lineages adapted to extreme climatic conditions show a pattern of increasing endemism because additional geographic factors appear to be acting as determinants of ancillary biome specialization.

### 4.3 Is DR related to biome specialization?

Biome specialists showed the highest mean DRs, while biome generalists presented decreasing DR as the number of inhabited biomes increases. Finally, extreme biome‐generalists (those occupying five or more biomes) showed a large variation among species, related to the relatively small number of species in this category. Our results on DR are consistent with those retrieved from the HiSSE analyses that showed that biome specialists diversified faster than biome generalists (Table S3.7). In addition, HiSSE results showed the effect of a hidden variable on diversification. Thus, significant effects of other variables, abiotic (e.g. tectonic; Badgley et al., 2017) of biotic (e.g. hostplants; Allio et al., 2021; Muto‐Fujita et al., 2017), are expected.

It is important to note that estimating DRs from extant species phylogenies is challenging (Burin et al., 2019; Louca & Pennell, 2020). Even more because of the relative incompleteness of species sampling in some tropical regions. Additionally, estimation becomes particularly complex when the group is ancient because there is an increasing probability that any possible diversification episode may have been veiled due to extinction wiping out entire lineages (Marshall, 2017).

Vrba hypothesized that biomes located at the extremes of the climatic conditions would be more impacted by global climatic changes, increasing their fragmentation rates, and thus, favouring extinction and speciation events, with a net increase in diversification. Results from swallowtail butterflies show that steppe, tundra and tropical deciduous woodland biomes have the highest DRs among biome‐specialist swallowtails (Figure 4b; Table S3.6 in Supporting Information). Steppe and tundra biomes are mainly occupied by the genus *Parnassius*, which showed some of the highest DRs among swallowtail butterflies (Figure 3). Nevertheless, our estimation for the DR of rainforest specialists was not as high as in other extreme biomes (Figure 4b). This could be related to a biotic process, or to the fact that the sampling for rainforest species for the phylogeny of the family covers ~55% of all rainforest specialists (Figure 3). The sclerophyllous woodland and shrubland showed the lowest DR values among all biome‐specialist swallowtails. Most of these biome specialists occupy the Mediterranean area. This region, because of its geography consisting of several islands and peninsulas restricted northwards by high mountain ranges, could have acted as refugia for temperate species during the glacial maxima of the Pleistocene, favouring the development of endemism (Bilton et al., 1998; Todisco et al., 2012; Zinetti et al., 2013). Their low DR values could mean that some sclerophyllous woodlands specialists may be considered as relicts, survivors of once more speciose clades that suffered extinction events during the glacial–interglacial alternation (Brown, 1995; Condamine, Rolland, et al., 2018).

### 4.4 The effect of species distribution areas on diversification

Both Variance Partitioning and PGLS results showed a stronger relationship between swallowtail DRs and their degree of biome specialization in comparison to species area. Biome specialization is about twice as explanatory as distribution area when studying swallowtail DRs in both analyses (*R* ^2^ adjusted = .133 vs. *R* ^2^ adjusted = .066; Figure 5) and PGLS (*R* ^2^ = .810 vs. *R* ^2^ = .487). These results confirm that species with large areas can be young, and species with small areas can be old (Gaston, 2003) and can reflect that, although swallowtail species diversification might be somehow related to dispersal capabilities, it is much more related to niche adaptation. The differences in explanatory power observed between variance partition and PGLS approaches are most probably related to the phylogenetic nature of the PGLS, suggesting that the correlation between variables is stronger within clades, getting diluted when swallowtails are studied altogether. These results agree with previous works that found these age–area relationships among closely related species (Gaston, 2003).

## 5 CONCLUSIONS

The swallowtails lineage presents a greater number of biome specialists and a lower number of biome generalists than expected. We show that this pattern stems from differential diversification of lineages: substantially higher DRs were detected among biome specialists compared to biome generalists. We also found that biome specialists are unevenly distributed across biomes, suggesting that past fragmentation events shaped the degree of biomes specialization Overall, our results are consistent with the resource‐use hypothesis, which states that global climatic changes, and the hypothetically subsequent biome fragmentation, promote divergence and speciation events in biome‐specialist lineages. Distinguishing the role of biomes as a main barrier for species expansion, and thus a main constraint conditioning the evolutionary pathways of lineages, is an open question yet to be answered. New analyses and simulations need to be conducted to measure the role of dispersal, species climatic niche and biome fragmentation as key factors promoting isolation. Our results point out the relevance of future conservation policies to maintain the ecological and evolutionary diversity within this family. Special effort should be placed into the identification and preservation of areas including specialist species with significantly high DRs (extreme biomes, mountain ranges) because fragmentation of such areas under the current and future situation of global climatic change will continue to foster the diversification of the group.

## CONFLICT OF INTEREST

All authors declare that they have no conflicts of interest to disclose.

## Supporting information


Data S1

## Data Availability

The R script used to analyse the data is available in GitHub (https://github.com/paleobicha/Papilionidae_Biome_Diversification_Analyses). The database containing detailed information of biome occupancy for each species is available in the Dryad Digital Depository (https://doi.org/10.5061/dryad.sbcc2fr5b).
