# Supplementary material for: A phylogenetic study to assess the link between biome specialization and diversification in swallowtail butterflies
Source: Glob Chang Biol. 2022 Jul 23;28(20):5901–13. doi: 10.1111/gcb.16344 (PMC9543414; doi:10.1111/gcb.16344)
Supplement: Supplementary file 1 — Data S1 [file GCB-28-5901-s001.pdf]

Supporting information

Appendix S1

Systematic references

Allio, R., Nabholz, B., Wanke, S., Chomicki, G., Pérez-Escobar, O. A., Cotton, A. M., Clamens, A., Kergoat, G. J., Sperling, F. A. H. & Condamine, F. L. (2021). Genome-wide macroevolutionary signatures of key innovations in butterflies colonizing new host plants. *Nature Communications*, *12*, 354.

Braby, M. F., Trueman, J. W. H. & Eastwood, R. (2005). When and where did troidine butterflies (Lepidoptera: Papilionidae) evolve? Phylogenetic and biogeographic evidence suggests an origin in remnant Gondwana in the Late Cretaceous. *Invertebrate Systematics, 19*, 113-143.

Caterino, M. S., Reed, R. D., Kuo, M. M. & Sperling, F. A. H. (2001). A partitioned likelihood analysis of swallowtail butterfly phylogeny (Lepidoptera: Papilionidae). *Systematic Biology, 50*, 106-127.

Condamine, F. L., Sperling, F. A., Wahlberg, N., Rasplus, J. Y. & Kergoat, G. J. (2012). What causes latitudinal gradients in species diversity? Evolutionary processes and ecological constraints on swallowtail biodiversity. *Ecology letters, 15*(3), 267-277.

Hancock, D. L. (1983). Classification of the Papilionidae (Lepidoptera): a phylogenetic approach. *Smithersia, 2*, 1-48.

Michel, F., Rebourg, C., Cosson, E. & Decimon, H. (2008). Molecular phylogeny of Parnassiinae butterflies (Lepidoptera: Papilionidae) based on the sequences of four mitochondrial DNA segments. *Annales de la Société Entomologique de France, 44*, 1–36.

Nazari, V., Zakharov, E. V. & Sperling, F. A. (2007). Phylogeny, historical biogeography, and taxonomic ranking of Parnassiinae (Lepidoptera, Papilionidae) based on morphology and seven genes. *Molecular phylogenetics and evolution, 42*(1), 131-156.

Silva-Brandão, K. L., Freitas, A. V. L., Brower, A. V. Z. & Solferini, V. N. (2005). Phylogenetic relationships of the New World Troidini swallowtails (Lepidoptera: Papilionidae) based on COI, COII, and EF-1α genes. *Molecular Phylogenetics and Evolution, 36*, 468-483.

Simonsen, T. J., Zakharov, E. V., Djernaes, M., Cotton, A. M., Vane-Wright, R. I. & Sperling, F. A. (2011). Phylogenetics and divergence times of Papilioninae (Lepidoptera) with special reference to the enigmatic genera *Teinopalpus* and *Meandrusa*. *Cladistics, 27*(2), 113-137.

Zakharov, E. V., Caterino, M. S. & Sperling, F. A. H. (2004). Molecular phylogeny, historical biogeography, and divergence time estimates for swallowtail butterflies of the genus *Papilio* (Lepidoptera: Papilionidae). *Systematic Biology, 53*, 193-215.

Table S1.1 Taxonomic reconciliation table including the decisions made to include or exclude some described taxa from our study and the references on which the decisions were based.

Taxonomic reconciliation	Reference
Graphium deliae (Libert & Collins, 2007) was included.	Libert, M. (2007) Note on the Genus *Grahpium* Scopoli. LambillioneaLibert, M. (2007). Note on the genus Graphium Scopoli. (Lepidoptera: Papilionidae). Lambillionea, 107(1), 19–29. CVII: 1-11
Papilio bacelarae (Bivar de Sousa & Mendez, 2009) was included.	Bivar de sousa, A. & Mendes, L. F. (2009). On a new species of the genus *Princeps* Hübner, [1807] from Cabinda (Angola) (Lepidoptera: Papilionidae). *SHILAP Revista de Lepidopterologia, 37*(147), 327-334.
Papilio rumiko, described in 2014, has been added separately from *Papilio cresphontes*.	Shiraiwa, K., Cong, Q., & Grishin, N. V. (2014). A new Heraclides swallowtail (Lepidoptera, Papilionidae) from North America is recognized by the pattern on its neck. *Zookeys*, (468), 85.
Parnassius huberi (Paulus, 1999) was considered.	Paulus, V. (1999). A new species of Parnassius discovered in North Tibet, China (Lepidoptera: Papilionidae, Parnassinae). *Wallace*, *6*, 1-7.
Graphium incertus is synonym of *Graphium parus*.	Racheli, T. & Cotton, A.M. (2009) Guide to the Butterflies of the Palearctic Region. Papilionidae. Part I. Omnes Artes, Milano,70 pp
Graphium mandarinus is synonym of *Graphium glycerion*.	Collins, N. M., & Morris, M. G. (1985). *Threatened swallowtail butterflies of the world: the IUCN Red Data Book*. Iucn.
Graphium timur (Ney, 1911) was synonymized with Graphium mullah following Cotton y Racheli, T., 2007.	Cotton, A.M. & Racheli, T., [2007]. A preliminary annotated checklist of the Papilionidae of Laos with notes on taxonomy, phenology, distribution and variation (Lepidoptera, Papilionoidea). Fragm. Entomol. 38 (2): 279-378
Iphiclides feisthamelli was considered as a separeted species from *Iphiclides podalirius*	Gaunet, A., Dincă, V., Dapporto, L., Montagud, S., Vodă, R., Schär, S., Badiane, A., Font, E. & Vila, R. (2019). Two consecutive Wolbachia-mediated mitochondrial introgressions obscure taxonomy in Palearctic swallowtail butterflies (Lepidoptera, Papilionidae). *Zoologica Scripta*, *48*(4), 507-519.
Luehdorfia longicaudata was considered as a separeted species from *Luehdorfia taibai*.	Lian-Xi, X., Peng-Fei, L., Jia, W., Kai, W., & Ping, Y. (2014). The complete mitochondrial genome of the endangered butterfly Luehdorfia taibai Chou (Lepidoptera: Papilionidae).
Ornithoptera arfakensiswas considered as a separeted species from *O.paradisea*	Adam Cotton personal communication/ Matsuka, H. (2001). *Natural history of birdwing butterflies*. Matsuka Shuppan.
Papilio appalachiensis was considered different from both *P. glaucus* and *P. canadiensis*	Kunte, K., Shea, C., Aardema, M. L., Scriber, J. M., Juenger, T. E., Gilbert, L. E., & Kronforst, M. R. (2011). Sex chromosome mosaicism and hybrid speciation among tiger swallowtail butterflies. *PLoS genetics*, *7*(9), e1002274.
Papilio daedalus was considered different from *Papilio palinurus*, and restricted to Philippines Islands.	Hardy, P. B., & Lawrence, J. M. (2017). *Field guide to butterflies of the Philippines* (p. 488). Siri Scientific Press. / Condamine, F. L., Toussaint, E. F., Cotton, A. M., Genson, G. S., Sperling, F. A., & Kergoat, G. J. (2013). Fine-scale biogeographical and temporal diversification processes of peacock swallowtails (Papilio subgenus Achillides) in the Indo-Australian Archipelago. Cladistics, 29(1), 88-111.
Papilio dehaani was considered different from *Papilio bianor*, and restricted to the japanese island of Hokkaido	East, R. F., Dubatolov, V. V., & Tshistjakov, Y. A. Papilio (Achillides) dehaanii C. et R. Felder, 1864 (Lepidoptera, Papilionidae) from the continental part of the of the Russian Far East. Euroasian Entomological Journal 20(2): 82-85.
Papilio enganius was considered different from *Papilio helenus*, and distributed in Enggano, Borneo and Lombok.	Allio, R., Nabholz, B., Wanke, S., Chomicki, G., Pérez-Escobar, O. A., Cotton, A. M., Clamens, A-L., Kergoat, G. J., Sperling, F. A. H. & Condamine, F. L. (2021). Genome-wide macroevolutionary signatures of key innovations in butterflies colonizing new host plants. *Nature communications*, *12*(1), 1-15.

Papilio heringi was considered different from *Papilio fuscus*, and endemic from Halmahera Island.	MOONEN, J. (1996). A new natural hybrid of Papilio Linnaeus from W. Irian and remarks on Papilio heringi Niepelt from Halmahera (Lepidoptera, Papilionidae). *Lepidoptera Science*, *47*(3), 185-188.
Papilio hermeli was considered different from *Papilio chikae*, endemic from Mindoro Island	Hardy, P. B., & Lawrence, J. M. (2017). Field guide to butterflies of the Philippines (p. 488)
Papilio hippocrates was considered different from *Papilio machaon*	Remington, C. L. (1960). Wide experimental crosses between Papilio xuthus and other species. *J Lep Soc*, *13*, 151-164.
Papilio humbloti was considered different from *Papilio dardanus*, endemic to Comoro Islands	Allio, R., Nabholz, B., Wanke, S., Chomicki, G., Pérez-Escobar, O. A., Cotton, A. M., Clamens, A-L., Kergoat, G. J., Sperling, F. A. H. & Condamine, F. L. (2021). Genome-wide macroevolutionary signatures of key innovations in butterflies colonizing new host plants. *Nature communications*, *12*(1), 1-15.
Papilio meriones was considered a different species from *Papilio dardanus*, endemic to Madagascar	Allio, R., Nabholz, B., Wanke, S., Chomicki, G., Pérez-Escobar, O. A., Cotton, A. M., Clamens, A-L., Kergoat, G. J., Sperling, F. A. H. & Condamine, F. L. (2021). Genome-wide macroevolutionary signatures of key innovations in butterflies colonizing new host plants. *Nature communications*, *12*(1), 1-15.
Papilio oviedo was considered as a separeted species from *Papilio thoas*, endemic to Cuba	AGUILA, R. N., & CAÑAMERO, A. B. (2012). A list of Cuban Lepidoptera (Arthropoda: Insecta). *Zootaxa*, *3384*(1), 1-59.
Papilio pallas was considered different from *P. astyalus.*	Allio, R., Nabholz, B., Wanke, S., Chomicki, G., Pérez-Escobar, O. A., Cotton, A. M., Clamens, A-L., Kergoat, G. J., Sperling, F. A. H. & Condamine, F. L. (2021). Genome-wide macroevolutionary signatures of key innovations in butterflies colonizing new host plants. *Nature communications*, *12*(1), 1-15.
Papilio polyctor was considered as a separeted species from *Papilio bianor*	Lixin, Z., Xiaobing, W., Chunsheng, W., & Banghe, Y. (2009). Phylogenetic evaluation of Papilio bianor and P. polyctor (Lepidoptera: Papilionidae). *Oriental Insects*, *43*(1), 25-32.
Papilio prexaspes was considered different fromPapilio fuscus.	COTTON, A. M., & RACHELI, T. (2006). Notes on some Papilionidae of Laos and Vietnam. *Fragmenta entomologica*, *38*(1), 145-154.
Parnassius augustus was considered a different species from Parnassius imperator	Omoto, K., Katoh, T., Chichvarkhin, A., & Yagi, T. (2004). Molecular systematics and evolution of the "Apollo" butterflies of the genus Parnassius (Lepidoptera: Papilionidae) based on mitochondrial DNA sequence data. *Gene*, *326*, 141-147.
Parnassius jacobsoni and Parnassius cardinal were considered separated species from Parnassius staudingeri.	Korb, S. K. (2020). An annotated checklist of the tribus Parnassiini sensu Korshunov of the Old World (Lepidoptera, Papilionidae). *Acta Biologica Sibirica*, *6*, 59.
Parnasius mercurius was considered different P. jacquemontii	Korb, S. K. (2020). An annotated checklist of the tribus Parnassiini sensu Korshunov of the Old World (Lepidoptera, Papilionidae). *Acta Biologica Sibirica*, *6*, 59.

Appendix S2

Literature used for the biogeographical survey of all modern Papilionidae species*

* In those cases in which two or more references contained geographical information for a species, the most recent information has always been taken.

Acharya, B. K. & Vijayan, L. (2011). Butterflies of Sikkim with reference to elevational gradient in species, abundance, composition, similarity and range size distribution. In: L. Arrawatia & S. Tambe. (Eds.), *Biodiversity of Sikkim: Exploring and Conserving a Global Hotspot* (pp. 207-220) Sikkim: Information and Public Relations Department, Government of Sikkim.

Ackery, P. R. (1975). Guide to the genera and species of Parnassiinae (Lepidoptera: Papilionidae). *Bulletin of the British Museum of Natural History: Entomology, 31*, 71-105.

Andrade-C, M. G. (2002). Biodiversidad de las mariposas (Lepidoptera: Rhopalocera) de Colombia. *Boletín de la Sociedad Entomológica Aragonesa, 2*, 153-172.

Anto, M., Binoy, C. F., & Anto, I. (2021). Endemism-based butterfly conservation: insights from a study in Southern Western Ghats, India. The Journal of Basic and Applied Zoology, 82(1), 1-19.

Armstrong, A.J. & Wallbank, R. (2020). Graphium antheus. The IUCN Red List of Threatened Species 2020: e.T160008A161325891. https://dx.doi.org/10.2305/IUCN.UK.2020-3.RLTS.T160008A161325891.en.

Armstrong, A.J. & Westrip, J.R.S. (2020). Graphium colonna. The IUCN Red List of Threatened Species 2020: e.T121978812A161331514. https://dx.doi.org/10.2305/IUCN.UK.2020-3.RLTS.T121978812A161331514.en.

Armstrong, A.J. & Westrip, J.R.S. (2020). Graphium morania. The IUCN Red List of Threatened Species 2020: e.T122058707A161330510. https://dx.doi.org/10.2305/IUCN.UK.2020-3.RLTS.T122058707A161330510.en.

Arora, G. S. & Mondal, D. K. (1981). *On the Papilioninae (Papilionidae, Lepidoptera) from Arunachal Pradesh & Adjoining Areas of Assam in Northeastern India*. New Delhi: Controller of Publications. 65 pp.

Bains, T. & Böhm, M. (2021). Cressida cressida. The IUCN Red List of Threatened Species 2021: e.T121975467A122602326. https://dx.doi.org/10.2305/IUCN.UK.2021-2.RLTS.T121975467A122602326.en.

Bains, T. & Moonen, J. (2021). Graphium codrus. The IUCN Red List of Threatened Species 2021: e.T121980998A122602501. https://dx.doi.org/10.2305/IUCN.UK.2021-2.RLTS.T121980998A122602501.en.

Bains, T. & Moonen, J. (2021). Graphium empedovana. The IUCN Red List of Threatened Species 2021: e.T121979208A122602441. https://dx.doi.org/10.2305/IUCN.UK.2021-2.RLTS.T121979208A122602441.en.

Bains, T. & Moonen, J. (2021). Graphium euphrates. The IUCN Red List of Threatened Species 2021: e.T121979293A122602456. https://dx.doi.org/10.2305/IUCN.UK.2021-2.RLTS.T121979293A122602456.en.

Bains, T., Moonen, J. & Müller, C.J. (2021). Graphium browni. The IUCN Red List of Threatened Species 2021: e.T121976901A122602406. https://dx.doi.org/10.2305/IUCN.UK.2021-2.RLTS.T121976901A122602406.en.

Bains, T., Moonen, J. & Müller, C.J. (2021). Graphium kosii. The IUCN Red List of Threatened Species 2021: e.T122057257A122602531. https://dx.doi.org/10.2305/IUCN.UK.2021-2.RLTS.T122057257A122602531.en.

Bains, T., Moonen, J., Müller, C.J. & Peggie, D. (2021). Graphium meyeri. The IUCN Red List of Threatened Species 2021: e.T161120A122601865. https://dx.doi.org/10.2305/IUCN.UK.2021-2.RLTS.T161120A122601865.en.

Bains, T., Moonen, J. & Peggie, D. (2021). Graphium sarpedon. The IUCN Red List of Threatened Species 2021: e.T160215A821002. https://dx.doi.org/10.2305/IUCN.UK.2021-2.RLTS.T160215A821002.en.

Bains, T., Moonen, J. & Peggie, D. (2021). Pachliopta polydorus. The IUCN Red List of Threatened Species 2021: e.T121973642A122602261. https://dx.doi.org/10.2305/IUCN.UK.2021-2.RLTS.T121973642A122602261.en.

Bains, T., Moonen, J., Peggie, D. & Müller, C.J. (2021). Papilio blumei. The IUCN Red List of Threatened Species 2021: e.T122524294A122602851. https://dx.doi.org/10.2305/IUCN.UK.2021-2.RLTS.T122524294A122602851.en.

Bains, T., Moonen, J., Peggie, D. & Müller, C.J. 2021. Papilio sataspes. The IUCN Red List of Threatened Species 2021: e.T122549231A122603331. https://dx.doi.org/10.2305/IUCN.UK.2021-2.RLTS.T122549231A122603331.en.

Bains, T., Moonen, J., Peggie, D. & Müller, C.J. (2021). Papilio veiovis. The IUCN Red List of Threatened Species 2021: e.T122549442A122603371. https://dx.doi.org/10.2305/IUCN.UK.2021-2.RLTS.T122549442A122603371.en.

Bains, T., Walker, A. & Hall, P. (2021). Battus philenor. The IUCN Red List of Threatened Species 2021: e.T110520147A110520288. https://dx.doi.org/10.2305/IUCN.UK.2021-1.RLTS.T110520147A110520288.en.

Ball, J. B., Geertsema, H., Samways, M. J., Henning, G. A. & Terblanche, R. F. (2009). Annotated list of the South African butterfly taxa with Red list assessments (with brief notes on the butterfly biota of South Africa, Lesotho and Swaziland. In: G. A. Henning, R. F. Terblanche & J. B. Ball (Eds.), *South African Red Data Book: Butterflies* (pp. 76-111). Pretoria: South African National Biodiversity Institute.

Bambaradeniya, C. N. (2006). *The Fauna of Sri Lanka: Status of Taxonomy, Research, and Conservation*. Gland: IUCN. 308 pp.

Barro, A. & Nuñez, R. (2011). *Lepidópteros de Cuba*. Vasa: UPC Print. 240 pp.

Bascombe, M. J., Johnston, G. & Bascombe, F. S. (1999). The Butterflies of Hong Kong. *Tropical Lepidoptera, 10*(2), 68.

Bauer, E. & Frankenbach, T. (1998). *Butterflies of the World: Papilionidae I: Achillides, Bhutanitis, Teinopalpus.* Keltern: Goecke & Evers. 20 pp.

Belcastro C. & Larsen, T. B. (2006). *Butterflies as an indicator group for the conservation value of the Gola Forests in Sierra Leone*. Freetown: Gola Forest Conservation Concession Project (GFCCP). 71 pp.

Benyamini, D., Ugarte, A., Shapiro, A. M., Mielk, O. H. H., Pyrcz, T. & Bálint, Z. (2014). An updated list of the butterflies of Chile (Lepidoptera, Papilionoidea and Hesperioidea) including distribution, flight period and conservation status part I, comprising the families: Papilionidae, Pieridae, Nymphalidae (in part) and Hesperiidae, describing a new species of Hypsochila (Pieridae) and a new subspecies of Yramea modesta (Nymphalidae). *Boletín del Museo Nacional de Historia Natural, 63*, 9-31.

Berends, A., Rosa, A., Mega, N., Marini-Filho, O. & Freitas, A.V.L. (2020). Parides orellana. The IUCN Red List of Threatened Species 2020: e.T122550072A122603451. https://dx.doi.org/10.2305/IUCN.UK.2020-2.RLTS.T122550072A122603451.en.

Berends, A., Rosa, A., Marini-Filho, O., Mega, N. & Freitas, A.V.L. (2020). Parides anchises. The IUCN Red List of Threatened Species 2020: e.T110618545A110618565. https://dx.doi.org/10.2305/IUCN.UK.2020-2.RLTS.T110618545A110618565.en

Bingham, C. T. (1901). *The Fauna of British India Including Ceylon and Burma: Butterflies (Vol. 1).* London: Taylor & Francis. 512 pp.

Bivar de sousa, A. (1983). Contribuicao para o conhecimento dos Lepidopteros de Angola (3.a nota). Dados sobre a ocorrencia do genero *Charaxes* (Lep., Nymphalidae) em Angola (1.a parte). *Actas del I Congreso Iberico de Entomologia, Facultad de Biologia, Leon, Spain, 7-10 June* (pp. 107-119). León: Servicio de publicaciones Universidad de León.

Bivar de sousa, A. & Mendes, L. F. (2009). On a new species of the genus *Princeps* Hübner, [1807] from Cabinda (Angola) (Lepidoptera: Papilionidae). *SHILAP Revista de Lepidopterologia, 37*(147), 327-334.

Bivar de Sousa, A., Consciencia, S. & Mendes, L. F. (2007). Novos dados sobre os lepidópteros diurnos (Lepidoptera: Hesperioidea e Papilionoidea) da Guiné-Bissau. II. Papilionidae e Pieridae. *Boletín de la Sociedad Entomológica Aragonesa, 41*, 223-236.

Böhm, M. (2018). Trogonoptera brookiana. The IUCN Red List of Threatened Species 2018: e.T91184152A91184305. https://dx.doi.org/10.2305/IUCN.UK.2018-1.RLTS.T91184152A91184305.en.

Böhm, M. 2019. Graphium macfarlanei. The IUCN Red List of Threatened Species 2019: e.T122058051A122602561. https://dx.doi.org/10.2305/IUCN.UK.2019-1.RLTS.T122058051A122602561.en.

Böhm, M. (2020). Trogonoptera trojana (amended version of 2018 assessment). The IUCN Red List of Threatened Species 2020: e.T91184396A177264999. https://dx.doi.org/10.2305/IUCN.UK.2020-3.RLTS.T91184396A177264999.en.

Böhm, M. (2021). Papilio anactus. The IUCN Red List of Threatened Species 2021: e.T122523973A122602811. https://dx.doi.org/10.2305/IUCN.UK.2021-2.RLTS.T122523973A122602811.en.

Böhm, M. (2021). Protographium leosthenes. The IUCN Red List of Threatened Species 2021: e.T122598899A122603746. https://dx.doi.org/10.2305/IUCN.UK.2021-2.RLTS.T122598899A122603746.en.

Böhm, M. & Müller, C.J. (2018). Graphium meeki (errata version published in 2021). The IUCN Red List of Threatened Species 2018: e.T9474A202206582. https://dx.doi.org/10.2305/IUCN.UK.2018-2.RLTS.T9474A202206582.en.

Böhm, M. & Müller, C.J. (2018). Papilio toboroi (errata version published in 2021). The IUCN Red List of Threatened Species 2018: e.T16007A202206830. https://dx.doi.org/10.2305/IUCN.UK.2018-2.RLTS.T16007A202206830.en.

Bollino, M. & Racheli, T. (2012). *Butterflies of the World, Supplement 20: Parnassiinae (Partim), Parnassiini (Partim), Luehdorfiini, Zerynthiini (Lepidoptera: Papilionidae)*. Keltern: Goecke & Evers. 64 pp.

Bollino, M. & Sala, G. (2004). *Synopsis of* Papilio alexanor *Esper, 1799: an updated review of its taxonomy and biology*. Bologna: Natura Edizioni Scientifiche. 64 pp.

Braby, M. (2016). *The complete field guide to butterflies of Australia*. Clayton: Csiro Publishing. 400 pp.

Brattström, O. (2010). *Butterflies at Sapo National Park.* Whein town: Liberia Forestry Development Authority and FFI International. 16 pp.

Brock, J. P. & Kaufman, K. (2003). *Kaufman field guide to butterflies of North America*. Boston: Houghton Mifflin Harcourt. 392 pp.

Bustos, E. O. N. (2009). Mariposas diurnas (Lepidoptera: Papilionoidea y Hesperioidea) del Parque Nacional Iguazú, Provincia de Misiones, Argentina. *Tropical Lepidoptera Research, 19*(2), 71-81.

Carcasson, R. H. (1960). The swallowtail butterflies of East Africa (Lepidoptera, Papilionidae). *Journal of the East Africa Natural History Society, Special supplement No. 6*, 1-33.

Carcasson, R. H. (1964). A preliminary survey of the zoogeography of African butterflies. *African Journal of Ecology, 2*(1), 122-157.

Carcasson, R. H. (1981). *Collins handguide to the butterflies of Africa*. London: Collins. 188 pp.

Carpenter, G. D. H. (1934a). New or rare African butterflies. *Proceedings of the Royal Entomological Society of London, 9*, 12-14.

Carpenter, G. D. H. (1934b). Some notes on the northern islands of Lake Victoria. *The Journal of Animal* Ecology, *3*, 91-104.

Carpenter, G. D. H. (1935). The Rhopalocera of Abyssinia a faunistic study. *Transactions of the Royal Entomological Society of London, 83*, 313-447.

Chandra, V. (2021). Papilio natewa. The IUCN Red List of Threatened Species 2021: e.T151562583A151579117. https://dx.doi.org/10.2305/IUCN.UK.2021-1.RLTS.T151562583A151579117.en.

Chandra, K., Chaudhary, L. K., Singh, R. K. & Koshta, M. L. (2002). Butterflies of Pench Tiger Reserve, Madhya Pradesh. *Zoos' Print Journal, 17*(10), 908-909.

Chernov, Y. I. & Tatarinov, A. G. (2006). Butterflies (Lepidoptera, Rhopalocera) in the arctic fauna. *Entomological Review, 86*(7), 760-786.

Choi, S. W. & Kim, S. S. (2012). The past and current status of endangered butterflies in Korea. *Entomological science, 15*(1), 1-12.

Chou, I. (1994a). *Monographia Rhopalocerorum Sinensium, Monograph of Chinese butterflies, Vol. 1*. Zhengzhou: Henan Scientific & Technological Publishing House. 408 pp.

Chou, I. (1994b). *Monographia Rhopalocerorum Sinensium, Vol 2*. Zhengzhou: Henan Scientific and Technological Publishing House. 445 pp.

Chowdhury, S., Aich, U. & Dash, M.K. (2014). Checklist of butterfly fauna of Dinajpur, Bangladesh. *India Journal of Entomology and Zoology Studies, 2*(5), 156-159.

Churkin, S. V. (2006). A new species of *Parnassius* Latreille, 1804 from Kyrgyzstan (Lepidoptera, Papilionidae). *Helios, 7*, 142-158.

Clark, R. & Vogler, A. P. (2009). A phylogenetic framework for wing pattern evolution in the mimetic mocker swallowtail *Papilio dardanus*. *Molecular ecology, 18*(18), 3872-3884.

Clarke, C. A., Gordon, I. J., Smith, C. R. & Vane-Wright, R. I. (1991). Phylogenetic relationships of three African swallowtail butterflies, *Papilio dardanus*, *P. phorcas* and *P. constantinus*: new data from hybrids (Lepidoptera: Papilionidae). *Systematic Entomology, 16* (3), 257-273.

Clarke. C. A. & Sheppard, P. M. (1959). The genetics of *Papilio dardanus* Brown. I. Race cenea from South Africa. *Genetics*, *44*, 1347-1358.

Collins, N. M. & Morris, M. G. (1985). *Threatened swallowtail butterflies of the world: the IUCN Red Data Book*. Gland: IUCN. 401 pp.

Collins, S. C. (2015). *Butterflies of the World, Part 42: The ABRI Collections 1: Papilionidae*. Keltern: Goecke & Evers. 36 pp.

Condamine, F. L., Toussaint, E. F., Cotton, A. M., Genson, G. S., Sperling, F. A. H. & Kergoat, G. J. (2013). Fine-scale biogeographical and temporal diversification processes of peacock swallowtails (*Papilio* subgenus *Achillides*) in the Indo-Australian Archipelago. *Cladistics, 29*(1): 88-111.

Congdon, T. C. E., Bampton, I. and Collins, S. C. (2009). Some notes on the life histories and taxonomy of Afrotropical *Graphium* species (Lepidoptera: Papilionoidea: Papilionidae). *Metamorphosis, 20*(2): 44-63.

Coote, L. D. (2000). *CITES identification guide-butterflies: guide to the identification of butterfly species controlled under the Convention on International Trade in Endangered Species of Wild Fauna and Flora*. Ottawa: Environment Canada. 221 pp.

Cotton, A. M. & Racheli, T. (2006). A preliminary annotated checklist of the Papilionidae of Laos with notes on taxonomy, phenology, distribution and variation (Lepidoptera, Papilionoidea). *Fragmenta Entomologica, 38*(2), 279-378.

D'abrera, B. (1990). *Butterflies of the Holarctic Region. Part 1. Papilionidae, Pieridae, Danaidae & Satyridae (part. 1)*. Victoria: Hill House Publishers. 185 pp.

Davenport, T. R. B. (2002). *Endemic butterflies of the Albertine Rift – an annotated checklist*. Mbeya: The Wildlife Conservation Society. 14 pp.

Davies, P. M. H. & Barnes, M. J. C. (1991). The butterflies of Mauritius. *Journal of Research on the Lepidoptera, 30*(3-4), 145-161.

De Niceville, L. (1890). *The Butterflies of India, Burmah and Ceylon* (Vol. 3). Moscow: Ripol Classic Publishing Group. 503 pp.

de Silva, G. (2007). *Bradt Sri Lankan Wildlife: A Visitor's Guide*. Buckinghamshire: Bradt Travel Guides. 144 pp.

Demay, S. (2010). *Parides genus database*. Available at http://parides.genus.free.fr. (Accessed 2017/07/10).

Dodson, J. (2005). Butterfly fauna of the Mpenjati Nature Reserve, KwaZulu-Natal, South Africa. *Metamorphosis, 16*(2), 47-52.

Easton, E. R. & Pun, W. W. (1997). New records of butterflies from Macau, Southeast China. *Tropical Lepidoptera, 8*(2), 60-66.

Ebner, J. A. (1971). Some notes on the Papilionidae of Manus Island, New Guinea. *Journal of the Lepidopterists' Society, 25*, 73-80.

Emery, E. D. O., Brown Jr, K. S. & Pinheiro, C. E. (2006). The butterflies (Lepidoptera, Papilionoidea) of the Distrito Federal, Brazil. *Revista Brasileira de Entomologia, 50*(1), 85-92.

Emmel, T. C. & Larsen, T. B. (1997). Butterfly diversity in Ghana, West Africa. *Tropical Lepidoptera Research,* 8(3), 1-13.

Erhardt, A. (1985). *Wiesen und Brachland als Lebensraum fiir Schmetterlinge. Eine Fallstudie im Tavetsch (GR).* Basel: Birkhiiuser. 154 pp.

Evans, W. H. (1932). *The identification of the Indian butterflies. Madras: Bombay*. Madras: The Bombay Natural History Society. 300 pp.

Fatimah, A. (2006). *Butterflies of Malaysian Borneo: a pocket guide*. Sarawak: University Malaysia Sarawak. 130 pp.

Fernando, E., Hu, S. & Lo, P. (2020). Bhutanitis mansfieldi. The IUCN Red List of Threatened Species 2020: e.T2797A122599264. https://dx.doi.org/10.2305/IUCN.UK.2020-2.RLTS.T2797A122599264.en.

Fernando, E., Hu, S., Lo, P. & Moonen, J. (2019). Byasa hedistus. The IUCN Red List of Threatened Species 2019: e.T121972078A122602166. https://dx.doi.org/10.2305/IUCN.UK.2019-3.RLTS.T121972078A122602166.en.

Fernando, E., Jangid, A.K., Alwis, C., Chowdhury, S., Jayasinghe, H.D., Kehimkar, I., Kunte, K., Rajapakshe, S.S. & Tiple, A.D. (2020). Pachliopta hector (amended version of 2019 assessment). The IUCN Red List of Threatened Species 2020: e.T121972074A170543045. https://dx.doi.org/10.2305/IUCN.UK.2020-2.RLTS.T121972074A170543045.en.

Fernando, E., Jangid, A.K., Chowdhury, S., Kehimkar, I., Lo, P. & Moonen, J. (2020). Losaria coon (amended version of 2019 assessment). The IUCN Red List of Threatened Species 2020: e.T121971752A170538377. https://dx.doi.org/10.2305/IUCN.UK.2020-2.RLTS.T121971752A170538377.en.

Fernando, E., Jangid, A.K., Irungbam, J.S., Kehimkar, I., Khanal, B., Kunte, K., Lo, P., Moonen, J., Qureshi, A.A. & Shrestha, B.R. (2020). Byasa dasarada (amended version of 2019 assessment). The IUCN Red List of Threatened Species 2020: e.T121971963A170542475. https://dx.doi.org/10.2305/IUCN.UK.2020-2.RLTS.T121971963A170542475.en.

Fernando, E., Jangid, A.K., Irungbam, J.S., Kehimkar, I., Kunte, K. & Lo, P. (2020). Bhutanitis lidderdalii (amended version of 2019 assessment). The IUCN Red List of Threatened Species 2020: e.T121975380A170537293. https://dx.doi.org/10.2305/IUCN.UK.2020-2.RLTS.T121975380A170537293.en.

Fernando, E., Jangid, A.K., Kehimkar, I., Khanal, B., Lo, P., Moonen, J., Qureshi, A.A. & Shrestha, B.R. (2020). Byasa latreillei (amended version of 2019 assessment). The IUCN Red List of Threatened Species 2020:

e.T121972222A170541505. https://dx.doi.org/10.2305/IUCN.UK.2020-2.RLTS.T121972222A170541505.en.

Fernando, E., Jangid, A.K., Kehimkar, I., Khanal, B., Lo, P., Moonen, J., Racheli, T. & Shrestha, B.R. (2020). Byasa alcinous (amended version of 2019 assessment). The IUCN Red List of Threatened Species 2020: e.T121971564A170542047. https://dx.doi.org/10.2305/IUCN.UK.2020-2.RLTS.T121971564A170542047.en.

Fernando, E., Jangid, A.K., Kehimkar, I., Khanal, B., Lo, P., Moonen, J. & Shrestha, B.R. (2020). Byasa plutonius (amended version of 2019 assessment). The IUCN Red List of Threatened Species 2020: e.T121973620A170539068. https://dx.doi.org/10.2305/IUCN.UK.2020-2.RLTS.T121973620A170539068.en.

Fernando, E., Jangid, A.K., Kehimkar, I. & Kunte, K. (2020). Bhutanitis ludlowi (amended version of 2019 assessment). The IUCN Red List of Threatened Species 2020: e.T2796A170536676. https://dx.doi.org/10.2305/IUCN.UK.2020-2.RLTS.T2796A170536676.en.

Fernando, E., Jangid, A.K., Kehimkar, I., Kunte, K., Lo, P., Moonen, J. & Qureshi, A.A. (2020). Byasa polla (amended version of 2020 assessment). The IUCN Red List of Threatened Species 2020: e.T121973631A170546153. https://dx.doi.org/10.2305/IUCN.UK.2020-2.RLTS.T121973631A170546153.en.

Fernando, E., Jangid, A.K., Kehimkar, I., Kunte, K., Moonen, J. & Tiple, A.D. (2020). Pachliopta pandiyana (amended version of 2019 assessment). The IUCN Red List of Threatened Species 2020: e.T121973590A170544028. https://dx.doi.org/10.2305/IUCN.UK.2020-2.RLTS.T121973590A170544028.en.

Fernando, E., Lechner, K., Moonen, J. & Racheli, T. (2019). Atrophaneura adamsoni. The IUCN Red List of Threatened Species 2019: e.T121971502A122602111. https://dx.doi.org/10.2305/IUCN.UK.2019-3.RLTS.T121971502A122602111.en.

Fernando, E., Moonen, J. & Racheli, T. (2020). Byasa laos (amended version of 2019 assessment). The IUCN Red List of Threatened Species 2020: e.T121972215A176106795. https://dx.doi.org/10.2305/IUCN.UK.2020-3.RLTS.T121972215A176106795.en.

Fernando, E. & Walker, A. (2021). Papilio zelicaon. The IUCN Red List of Threatened Species 2021: e.T110618291A110618297. https://dx.doi.org/10.2305/IUCN.UK.2021-1.RLTS.T110618291A110618297.en.

Fox, R. M. (1963). New African butterflies. *Annals of the Carnegie Museum, 36*, 213-224.

Fox, R. M. (1965). *The butterflies of Liberia*. Philadelphia: American Entomological Society at the Academy of Natural Sciences. 438 pp.

Francini, R. B., Duarte, M., Mielke, O. H. H., Caldas, A. & Freitas, A. V. L. (2011). Butterflies (Lepidoptera, Papilionoidea and Hesperioidea) of the" Baixada Santista" region, coastal São Paulo, southeastern Brazil. *Revista Brasileira de Entomologia, 55*(1), 55-68.

Frankenbach, T., Bollino, M. & Racheli, T. (2012). *Butterflies of the World, Part 36: Papilionidae XIV: Hypermnestra, Luehdorfiini, Zerynthiini*. Keltern: Goecke & Evers. 29 pp.

Freitas, A. V. L. & Marini-Filho, O. J. (2011). *Plano de açao nacional para conservaçao dos lepidópteros ameaçados de extinçao*. Brasilia: ICMBio. 124 pp.

Gabriel, A. G. (1949). Notes on the Rhopalocera of Abyssinia. *Proceedings of the Royal Entomological Society of London B, 18*, 207-216.

García-Barros, E. (2004). *Atlas de las mariposas diurnas de la Península Ibérica e islas Baleares (Lepidoptera: Papilionoidea & Hesperioidea)*. Zaragoza: Sociedad Entomológica Aragonesa. 228 pp.

García-Robledo, C., Constantino, L. M., Heredia, M. D. & Kattan, G. (2002). *Mariposas comunes de la cordillera Central de Colombia*. Cali: Wildlife Conservation Society-Colombia. 130 pp.

Glassberg, J. (2018). *A Swift Guide to Butterflies of Mexico and Central America.* Princeton; Princeton University Press. 304 pp.

Glassberg, J., Minno, M. C. & Calhoun, J. V. (2000). *Butterflies through binoculars*. Oxford: Oxford University Press. 400 pp.

Gorbunov, P. I. & Kosterin, O. E. (2007). *The butterflies (Hesperioidea and Papilionoidea) of North Asia (Asian part of Russia) in nature* (Vol. 2). Moscow: Rodina & Fodio. 408 pp.

Gordon, I. & Cobblah, M. (2000). Insects of the Muni-Pomadze Ramsar site. *Biodiversity and Conservation, 9*(4), 479-486.

Gratton, P. (2006). *Phylogeography and conservation genetics of Parnassius mnemosyne L., 1758 (Lepidoptera, Papilionidae).* Published PhD Thesis, University of Rome 'Tor Vergata.

Grice, H. & Böhm, M. (2018). Eurytides serville. The IUCN Red List of Threatened Species 2018: e.T161102A873426. https://dx.doi.org/10.2305/IUCN.UK.2018-2.RLTS.T161102A873426.en.

Grice, H. & Böhm, M. (2018). Protographium anaxilaus. The IUCN Red List of Threatened Species 2018: e.T160184A818970. https://dx.doi.org/10.2305/IUCN.UK.2018-2.RLTS.T160184A818970.en.

Grice, H., Freitas, A.V.L., Mielke, O. & Casagrande, M. (2018). Heraclides chiansiades. The IUCN Red List of Threatened Species 2018: e.T122524581A122602876. https://dx.doi.org/10.2305/IUCN.UK.2018-2.RLTS.T122524581A122602876.en.

Grice, H., Freitas, A.V.L., Mielke, O. & Casagrande, M. (2018). Heraclides garleppi. The IUCN Red List of Threatened Species 2018: e.T15989A122599925. https://dx.doi.org/10.2305/IUCN.UK.2018-2.RLTS.T15989A122599925.en.

Grice, H., Hall, P., Freitas, A.V.L., Rosa, A., Dias, F.M.S., Marini-Filho, O. & Mega, N. (2019). Eurytides iphitas (amended version of 2018 assessment). The IUCN Red List of Threatened Species 2019: e.T8415A145165515. https://dx.doi.org/10.2305/IUCN.UK.2018-2.RLTS.T8415A145165515.en.

Grice, H., Freitas, A.V.L., Rosa, A., Dias, F.M.S., Mega, N., Marini-Filho, O., Casagrande, M. & Mielke, O. (2019). Eurytides bellerophon (amended version of 2018 assessment). The IUCN Red List of Threatened Species 2019: e.T121975575A145167045. https://dx.doi.org/10.2305/IUCN.UK.2019-1.RLTS.T121975575A145167045.en.

Grice, H., Freitas, A.V.L., Rosa, A., Marini-Filho, O., Mega, N., Dias, F.M.S., Casagrande, M. & Mielke, O. (2019). Eurytides callias (amended version of 2018 assessment). The IUCN Red List of Threatened Species 2019: e.T121975634A145167189. https://dx.doi.org/10.2305/IUCN.UK.2019-1.RLTS.T121975634A145167189.en.

Grice, H., Freitas, A.V.L., Rosa, A., Mega, N., Dias, F.M.S., Marini-Filho, O., Casagrande, M. & Mielke, O. (2019). Protographium asius (amended version of 2018 assessment). The IUCN Red List of Threatened Species 2019: e.T122598883A145167556. https://dx.doi.org/10.2305/IUCN.UK.2019-1.RLTS.T122598883A145167556.en.

Grice, H., Hall, P., Mega, N., Nunez-Bustos, E. & Freitas, A.V.L. (2018). Battus polydamas. The IUCN Red List of Threatened Species 2018: e.T110520503A110520514. https://dx.doi.org/10.2305/IUCN.UK.2018-2.RLTS.T110520503A110520514.en.

Grice, H., Marini-Filho, O., Freitas, A.V.L., Rosa, A., Dias, F.M.S. & Mega, N. (2019). Heraclides himeros (amended version of 2018 assessment). The IUCN Red List of Threatened Species 2019: e.T15991A145165665. https://dx.doi.org/10.2305/IUCN.UK.2019-1.RLTS.T15991A145165665.en.

Grice, H. & Nunez-Bustos, E. (2018). Battus madyes. The IUCN Red List of Threatened Species 2018: e.T121975252A122602311. https://dx.doi.org/10.2305/IUCN.UK.2018-2.RLTS.T121975252A122602311.en.

Grice, H., Nunez-Bustos, E., Casagrande, M., Mielke, O. & Freitas, A.V.L. (2018). Eurytides dolicaon. The IUCN Red List of Threatened Species 2018: e.T110524167A110524178. https://dx.doi.org/10.2305/IUCN.UK.2018-2.RLTS.T110524167A110524178.en.

Grice, H., Nunez-Bustos, E., Freitas, A.V.L., Rosa, A., Marini-Filho, O., Dias, F.M.S., Mega, N., Mielke, O. & Casagrande, M. (2019). Protographium agesilaus (amended version of 2018 assessment). The IUCN Red List of Threatened Species 2019: e.T110716929A145166699. https://dx.doi.org/10.2305/IUCN.UK.2018-2.RLTS.T110716929A145166699.en.

Grice, H., Nunez-Bustos, E., Mega, N., Casagrande, M., Mielke, O. & Freitas, A.V.L. (2018). Battus polystictus. The IUCN Red List of Threatened Species 2018: e.T121975368A122602316. https://dx.doi.org/10.2305/IUCN.UK.2018-2.RLTS.T121975368A122602316.en.

Grice, H., Nunez-Bustos, E., Mega, N., Dias, F.M.S., Rosa, A., Freitas, A.V.L. & Marini-Filho, O. (2019). Euryades corethrus (amended version of 2018 assessment). The IUCN Red List of Threatened Species 2019: e.T160549A145166527. https://dx.doi.org/10.2305/IUCN.UK.2019-1.RLTS.T160549A145166527.en.

Grice, H., Nunez-Bustos, E., Mega, N., Freitas, A.V.L., Rosa, A., Marini-Filho, O., Dias, F.M.S., Mielke, O. & Casagrande, M. (2019). Mimoides lysithous (amended version of 2018 assessment). The IUCN Red List of Threatened Species 2019: e.T122083281A145167346. https://dx.doi.org/10.2305/IUCN.UK.2019-1.RLTS.T122083281A145167346.en.

Grice, H., Nunez-Bustos, E., Mega, N., Rosa, A., Freitas, A.V.L., Dias, F.M.S., Marini-Filho, O., Casagrande, M. & Mielke, O. (2019). Euryades duponchelii (amended version of 2018 assessment). The IUCN Red List of Threatened Species 2019: e.T121975537A145166849. https://dx.doi.org/10.2305/IUCN.UK.2018-2.RLTS.T121975537A145166849.en.

Hancock, D. L. (2009). Relationships of the swallowtail Genera' *'Agehana'* Matsumura,'*Chilasa*' Moore and '*Eleppone*' Hancock (Lepidoptera: Papilionidae). *Australian Entomologist, 36*(1), 7-12.

Hancock, D.L. (1984). The *Princeps nireus* group of swallowtails (Lepidoptera: Papilionidae). Systematics, phylogeny and biogeography. *Arnoldia Zimbabwe, 9*(12), 181-215.

Haribal, M. (2000). *The Butterflies of Sikkim Himalaya and their natural history*. New Delhi: Natraj Publishers. 217 pp.

Haroon, S. A. M., Rafi, M. A. & Ahmad, T. (2014). Checklist and Species Habitat of Butterfly Fauna in Union Council Koaz Bahram Dheri Khyber Pakhtunkhwa Pakistan. *International Journal of Fauna and Biological Studies, 1*(3), 37-39.

Heath, A., Newport, M. A. & Hancock, D. (2002). *Butterflies of Zambia*. Nairoby: African Butterfly Research Institute & Lepidopterist's Society of Africa. 137 pp.

Hecq, J. (1975). *Papilio maesseni* Berger au Togo. *Lambillionea, 75*(1-2), 15-16.

Henderson, C. L. (2010). Butterflies, Moths, an Other Invertebrates of Costa Rica. Austin: University of Texas Press. 187 pp.

Henning, G. A. (1994). Butterflies of the Mariepskop area. *Metamorphosis, 5*(4), 162-168.

Herrera, J. (1972). Mariposas comunes a Chile y Perú (Lepidoptera, Rhopalocera). *Revista Peruana de Entomología, 15*, 72-74.

Higgins, L. G. & Riley, N. D. (1970). *A field guide to the butterflies of Britain and Europe.* London: HarperCollins Distribution Services. 384 pp.

Higgins, L. G., Hargreaves, B. & Lhonoré, J. (1991). *Guide complet des papillons d'Europe et d'Afrique du Nord*. Lonay: Delachaux et Niestlé. 382 pp.

Hill, M. J. & Monastyrskii, A. L. (1999). Butterfly fauna of protected areas in North and Central Vietnam: collections 1994-1997 (Lepidoptera, Rhopalocera). *Atalanta,* 29, 185-208.

Homziak, N. T. & Homziak, J. (2006). *Papilio demoleus* (Lepidoptera: Papilionidae): a new record for the United States, commonwealth of Puerto Rico. *Florida Entomologist, 89*(4), 485-488.

Houlihan, P. R., Marchant, N. C. & Harrison, M. E. (2012). *A Guide to the Butterflies of Sabangau*. Palangka Raya: The Orangutan Tropical Peatland Project. 39 pp.

Hu, S. J., Cotton, A. M., Condamine, F. L., Duan, K., Wang, R. J., Hsu, Y. F., Zhang, X. & Cao, J. (2018). Revision of *Pazala* Moore, 1888: the *Graphium (Pazala) mandarinus* (Oberthür, 1879) group, with treatments of known taxa and descriptions of new species and new subspecies (Lepidoptera: Papilionidae). *Zootaxa, 4441*(3), 401-446.

Hu, S. J., Zhang, X., Cotton, A. M. & Ye, H. (2014). Discovery of a third species of *Lamproptera* Gray, 1832 (Lepidoptera: Papilionidae). *Zootaxa, 3786*(4), 469-482.

Huang, H. & Xue, Y. P. (2004). A contribution to the butterfly fauna of southern Yunnan. *Neue Entomologische Nachrichten, 57*, 135-154.

Hudson, G. V. (1928). *The butterflies and moths of New Zealand.* Wellington: Hudson and Osborn. 386 pp.

Huxley, J. (1976). The coloration of *Papilio zalmoxis* and *P. antimachus*, and the discovery of Tyndall blue in butterflies. *Proceedings of the Royal Society of London B, 193*(1113), 441-453.

Inayoshi, Y. (2017). *A check list of butterflies in Indo-China (chiefly from Thailand, Laos and Vietnam).* Available at http://yutaka.it-n.jp/papi.html. (Accessed 2017/07/10).

Inomata, T. (1986). *Atlas of the Japanese butterflies*. Tokyo: Takeshobo. 499 pp.

Kasambe, R. (2018) *Butterflies of Western Ghats*. Published by author. 372 pp.

Kasambe, R. & Wadatkar, J. (2008). Butterfly fauna in and around Nagpur city of Maharashtra. *Indian Lepidoptera, 4*, 3-8.

Katbeh-Bader, A., Amr, Z. S. & Isma'el, S. (2003). The butterflies of Jordan. *Journal of Research on the Lepidoptera, 87*, 11-26

Kato, Y. & Yagi, T. (2004). Biogeography of the subspecies of *Parides (Byasa) alcinous* (Lepidoptera: Papilionidae) based on a phylogenetic analysis of mitochondrial ND5 sequences. *Systematic Entomology, 29*(1), 1-9.

Kehimkar, I. D. (2008). *Book of Indian butterflies*. Oxford: Oxford University Press. 497 pp.

Khanal, B., Chalise, M. K., & Solanki, G. S. (2013). Threatened butterflies of central Nepal. *Journal of Threatened Taxa, 5*(11), 4612-4615.

Kielland, J. (1990). *Butterflies of Tanzania*. Melbourne: Hill House. 363 pp.

Kimura, Y., Aoki, T., Yamaguchi, S., Uemura, Y. & Saito, T. (2011). *The Butterflies of Thailand. Vol 1. Hesperiidae, Papilionidae and Pieridae*. Tokyo: Mokuyo-sha. 218 pp.

Kirton. L. G. (2014). *A Naturalist's Guide to the Butterflies of Peninsular Malaysia, Singapore and Thailand*. Oxford: John Beaufoy Publishing. 176 pp.

Kunte, K. (2000). *India, a Lifescape: Butterflies of Peninsular India*. Telangana: Universities Press. 254 pp.

Kunte, K. (2008). The Wildlife (Protection) Act and conservation prioritization of butterflies of the Western Ghats, southwestern India. *Current Science, 94*(6), 729-735.

Kunte, K., Sondhi, S., Sangma, B. M., Lovalekar, R., Tokekar, K. & Agavekar, G. (2012). Butterflies of the Garo Hills of Meghalaya, northeastern India: their diversity and conservation. *Journal of Threatened Taxa, 4*(10), 2933-2992.

Lafontaine, J. D. & Wood, D. M. (1997). Butterflies and moths (Lepidoptera) of the Yukon. In: H. V. Danks & J. A. Downes (Eds.), *Insects of the Yukon* (pp. 723-785). Otawa: Biological survey of Canada (terrestrial arthropods).

Lamas, G. (2004). *Atlas of Neotropical Lepidoptera: Checklist Part 4ª Hesperioidea-Papilionoidea*. Jodhpur: Scientific Publishers. 439 pp

Larsen, T. B. (1980a). *Butterflies of Oman*. Bartholomew Books, Edinburgh. 80 pp.

Larsen, T. B. (1980b). The status of *Papilio machaon rathjensi* and its relationship to other Arabian populations (Papilionidae). *Journal of the Lepidopterists' Society, 34*(4), 365-367.

Larsen, T. B. (1984). The zoogeographical composition and distribution of the Arabian butterflies (Lepidoptera; Rhopalocera). *Journal of Biogeography, 11*(2), 119-158.

Larsen, T. B. (1990). *The butterflies of Egypt*. Skerninge: Apollo Books. 112 pp.

Larsen, T. B. (1991). *The butterflies of Kenya and their natural history*. Oxford: Oxford University Press. 640 pp.

Larsen, T. B. (1992a). Butterfly collecting in the Tswapong Hills, Botswana (September, 1991). *Metamorphosis, 3*(1), 18-20.

Larsen, T. B. (1992b). The butterflies of the Gemsbok National Park in Botswana (Lepidoptera - Rhopalocera*). Botswana Notes and Records, 24*, 181-203.

Larsen, T. B. (1994). *The butterflies of Ghana – their implications for conservation and sustainable use*. Gland: Report to the IUCN and the Ghana Department of Game and Wildlife. 45 pp.

Larsen, T. B. (2004). *Butterflies of Bangladesh: An Annotated Checklist*. IUCN, the World Conservation Union, Bangladesh Country Office, Dhaka. 158 pp.

Larsen, T. B. (2008). Forest butterflies in West Africa have resisted extinction… so far (Lepidoptera: Papilionoidea and Hesperioidea). *Biodiversity and Conservation, 17*, 2833-2847.

Larsen, T. B., Riley, J. & Cornes, M. A. (1980). The butterfly fauna of a secondary bush locality in Nigeria. *Journal of Research in Lepidoptera, 18*, 4-23.

Layberry, R. A., Hall, P. W. & Lafontaine, J. D. (1998). *The butterflies of Canada*. Toronto: University of Toronto Press. 354 pp.

Le Crom, J., Constantino, L. & Salazar, J. (2002). *Butterflies of Colombia, Papilionidae*. Bogotá: Carlec. 122 pp.

Leech, J. H. (1888). On the Lepidoptera of Japan and Corea. *Proceedings of the Zoological Society of London, 56*(1), 580-655.

Lewis, D. S. A. (2010). *Phylogeny and Revision of the Genus Heraclides Hübner, 1819 (Lepidoptera: Papilionidae: Papilioninae: Papilionini)* [Doctoral dissertation, University of Florida, Gainesville]. 301 pp.

Li, X. (2019). Bhutanitis thaidina. The IUCN Red List of Threatened Species 2019: e.T2798A122599287. https://dx.doi.org/10.2305/IUCN.UK.2019-3.RLTS.T2798A122599287.en.

Li, X., Zhang, Y., Luo, Y. & Settele, J. (2006). Life history, life table, habitat, and conservation of *Byasa impediens* (Lepidoptera: Papilionidae). *Acta Ecologica Sinica, 26*(10), 3184-3197.

Libert, M. (2007). Note on the genus *Graphium* Scopoli. (Lepidoptera: Papilionidae). *Lambillionea, 107*(1), 19–29.

Llorente-Bousquets, J. (1997). *Papilionidae y Pieridae de México: Distribución geográfica e ilustración*. [Doctoral dissertation, Universidad Nacional Autonoma de México, Ciudad de México]. 226 pp.

Llorente-Bousquets, J., Vargas-Fernández, I., Luis-Martínez, A., Trujano-Ortega, M., Hernández-Mejía, B. C. & Warren, A. D. (2014). Biodiversidad de Lepidoptera en México. *Revista mexicana de biodiversidad, 85*, 353-371.

Maes, J. M. (2006). Papilionidae (Lepidoptera) de Nicaragua. *Revista Nicaragüense de Entomología, 66*(Supl. 3), 1-241.

Mahendra, K., Manish, K. & Vivek, K. (2013). Diversity of Butterflies (Lepidoptera) in Bilaspur district, Chhattisgarh, India. *Asian Journal of Experimental Biological Sciences, 4*(2), 282-287.

Mani, M. S. (2012). *Ecology and biogeography in India*. Berlin: Springer Science & Business Media. 773 pp.

Martin, I. (2015). Preliminary checklist and state of conservation of butterflies (Lepidoptera: Papilionoidea) of the Caldera de Luba, Bioko Island, Equatorial Guinea. *African Entomology, 23*(2), 376-386.

Martín, I. & Cobos, P. (2016). Rhopalocera de la Caldera de Lubá, isla de Bioko (Guinea Ecuatorial): Papilionidae, Pieridae y Lycaenidae (Lepidoptera: Papilionoidea). *SHILAP Revista de Lepidopterología, 44*(173), 157-168.

Martínez, A., Pozo, C. & May, E. (2005). Las mariposas (Rhopalocera: Papilionidae, Pieridae y Nymphalidae) de la selva alta subperennifolia de la región de Calakmul, México, con nuevos registros. *Folia Entomológica Mexicana, 44*(2), 123-143.

Mendes, L. F., Bivar-de-Sousa, A. & Williams, M. C. (2019). The Butterflies and Skippers (Lepidoptera: Papilionoidea) of Angola: An Updated Checklist. In: B. J. Huntley, V. Russo, F. Lages & N. Ferrand, N. (Eds.), *Biodiversity of Angola* (pp. 167-203). Cham: Springer.

Mohanraj, P. & Veenakumari, K. (1996). Host plants, phenologies and status of swallowtails (Papilionidae), Lepidoptera, in the Andaman and Nicobar Islands, Bay of Bengal, Indian Ocean. *Biological conservation, 78*(3), 215-221.

Möhn, E. (1999a). *Butterflies of the World, Part 5: Papilionidae II:* Battus. Keltern: Goecke & Evers. 20 pp

Möhn, E. (1999b). *Butterflies of the World, Part 8: Papilionidae V: New and Rare Papilionidae*. Keltern: Goecke & Evers. 9 pp

Möhn, E. (2003*). Butterflies of the World, Part 13: Papilionidae VII:* Parnassius apollo *I Plates*. Keltern: Goecke & Evers. 35 pp

Möhn, E. (2005). *Butterflies of the World, Part 23: Papilionidae XII:* Parnassius apollo *III*. Keltern: Goecke & Evers. 33 pp

Möhn, E. (2007). *Butterflies of the World, Part 26: Papilionidae XIII:* Parides. Keltern: Goecke & Evers. 52 pp.

Möhn, E., Bauer, E. & Frankenbach, T. (2002). *Butterflies of the World, Part 14: Papilionidae VIII: Baronia, Euryades, Protographium, Neographium, Eurytides*. Keltern: Goecke & Evers. 48 pp

Monastyrskii, A. L. (2007). *Butterflies of Vietnam. Volume 2. Papilionidae.* Apollo Books. 189 pp.

Monastyrskii, A. L. & Holloway, J. D. (2013). The Biogeography of the Butterfly Fauna of Vietnam With a Focus on the Endemic Species (Lepidoptera). In: M. Silva-Opps (Ed.), *Current progress in biological research* (pp. 95-123). Rijeka: INTECH Open Access Publisher.

Motta, P. C. (2002). Butterflies from the Uberlândia region, central Brazil: species list and biological comments. *Brazilian Journal of Biology, 62*(1), 151-163.

Müller, C.J. & Böhm, M. (2018). Papilio weymeri (errata version published in 2021). The IUCN Red List of Threatened Species 2018: e.T16008A202206937. https://dx.doi.org/10.2305/IUCN.UK.2018-2.RLTS.T16008A202206937.en.

Müller, C. J. & Tennent, W. J. (1999). A new species of *Graphium* Scopoli (Lepidoptera: Papilionidae) from the Bismarck Archipelago, Papua New Guinea. *Records of the Australian Museum, 51*, 161-168.

Nadler, J., Benyamini, D., Bonelli, S., Comay, O., Dapporto, L., Karaçetin, E., Lukhtanov, V., López Munguira, M., Micevski, N., Settele, J., Tzortzakaki, O., Verovnik, R., Warren, M., Wiemers, M., Wynhoff, I. & van Swaay, C. (2021). Parnassius mnemosyne. The IUCN Red List of Threatened Species 2021: e.T174210A122602056. https://dx.doi.org/10.2305/IUCN.UK.2021-1.RLTS.T174210A122602056.en.

Nadler, J., Bonelli, S., Dapporto, L., Karaçetin, E., Lukhtanov, V., López Munguira, M., Micevski, N., Settele, J., Tzortzakaki, O., Verovnik, R., Warren, M., Wiemers, M., Wynhoff, I. & van Swaay, C. 2021. Parnassius apollo. The IUCN Red List of Threatened Species 2021: e.T16249A122600528. https://dx.doi.org/10.2305/IUCN.UK.2021-1.RLTS.T16249A122600528.en.

Nadler, J. & Lukhtanov, V. (2020). Parnassius stubbendorfii. The IUCN Red List of Threatened Species 2020: e.T122553272A122603696. https://dx.doi.org/10.2305/IUCN.UK.2020-2.RLTS.T122553272A122603696.en.

Nadler, J. & Lukhtanov, V. (2021). Parnassius autocrator. The IUCN Red List of Threatened Species 2021: e.T16251A122600766. https://dx.doi.org/10.2305/IUCN.UK.2021-1.RLTS.T16251A122600766.en.

Nadler, J. & Lukhtanov, V. (2021). Parnassius tenedius. The IUCN Red List of Threatened Species 2021: e.T122553328A122603706. https://dx.doi.org/10.2305/IUCN.UK.2021-1.RLTS.T122553328A122603706.en.

Nadler, J., Lukhtanov, V., Hall, P. & Shuey, J. (2021). Parnassius eversmanni. The IUCN Red List of Threatened Species 2021: e.T110619537A110619548. https://dx.doi.org/10.2305/IUCN.UK.2021-1.RLTS.T110619537A110619548.en.

Nakae, M. (2021). *Papilionidae of the World. Tokyo:* Roppon-Ashi Entomological Books. 336 pp.

Nakanishi, A., Jalil, M. F. & Wahid, N. (2004). *Catalogue of Swallowtail Butterflies (lepidoptera: Papilionidae) at BORNEENSIS*. Sabah: Research & Education Component, Bornean Biodiversity and Ecosystem Conservation (BBEC) Programme in Sabah c/o Institute for Tropical Biology and Conservation (ITBC), University Malaysia. 42 pp.

Nishimura, M. (1996). Notes on some Papilionidae from Indo-China (2). *Transactions of the Lepidopterological Society of Japan, 47*(1), 40-48.

Ohya, T. (2009). *Catalogue of Birdwing Butterflies* (Revised edition). Buckinghamshire: Pemberley Books. 288 pp.

Orr, A. & Kitching, R. (2010). *The butterflies of Australia*. Crows Nest: Allen & Unwin. 328 pp.

Osada, S., Uemura, Y. & Uehara, J. (1999). An Illustrated Checklist of the Butterflies of Laos P.D.R. Tokyo: Mokuyo-sha. 240 pp.

Özden, Ö. (2013). Habitat preferences of butterflies (Papilionoidea) in the Karpaz Peninsula, Cyprus. *Nota Lepidopterologica, 36*(1), 57-64.

Padhye, A., Shelke, S. & Dahanukar, N. (2012). Distribution and composition of butterfly species along the latitudinal and habitat gradients of the Western Ghats of India. *Check List, 8*(6), 1197-1215.

Page, M. G. P., Treadaway, C. G., Bauer, E. & Frankenbach, T. (2003). *Butterflies of the World, Part 17: Papilionidae IX: Papilionidae of the Philippine Islands*. Keltern: Goecke & Evers. 71 pp.

Parsons, M. J. (1996). The immature stages of *Pharmacophagus antenor* (Drury) (Papilionidae: Troidini) from Madagascar. *Journal of the Lepidopterists Society, 50*, 337-344.

Patil, K. G. (2014). Butterfly diversity of Gorewada International Bio-Park, Nagpur, Central India. *Arthropods, 3*(2), 111-119.

Patrick, B. & Chandra, V. (2019). Papilio schmeltzi. The IUCN Red List of Threatened Species 2019: e.T160879A859235. https://dx.doi.org/10.2305/IUCN.UK.2019-3.RLTS.T160879A859235.en.

Patrick, B., Edwards, E. & Böhm, M. (2018). Papilio godeffroyi. The IUCN Red List of Threatened Species 2018: e.T122530997A122603026. https://dx.doi.org/10.2305/IUCN.UK.2018-2.RLTS.T122530997A122603026.en.

Penney, D. (2009). *Field Guide to the Butterflies of The Gambia, West Africa*. Manchester: Siri Scientific Press. 80 pp.

Pérez Ruiz, H. (1977). Distribución geográfica y estructura poblacional de *Baronia brevicornis* Salv. (Lepidoptera, Papilionidae, Baroniinae) en la República Mexicana. *Anales del Instituto Biología de la Universidad Nacional Autónoma de* México, *48*, 151-164

Perveen, F. (2012). Distribution of butterflies (Lepidoptera) of Kohat, Khyber Pakhtunkhwa, Pakistan. *Agricultural Science Research Journal*, *2*(9), 539-549.

Pinhey, E. & Loe, I. (1977). *A guide to the butterflies of Zambia*. London: Anglo American Corporation. 106 pp.

Pittaway, A. R., Larsen, T. B., Clarke, C. A., Smith, C. R., Crnjar, R. & Clarke, F. M. M. (1994). *Papilio saharae* Oberthur, 1879, specifically distinct from *Papilio machaon* Linnaeus, 1758 (Lepidoptera: Papilionidae). *Entomologist's Gazette, 45*(4), 223-249.

Poel, P. Van der & Wangchuk, T. (2007). *Butterflies of Bhutan. Mountains, hills and valleys between*, *800 and 3000m*. Timphu: Royal Society for the Protection of Natur. 71pp.

Poulton, E. B. (1925). Interesting new races of Papilios from S.W. Abyssinia and Somaliland. *Proceedings of the Entomological Society of London, 1925*, 42-52.

Pringle, E. L. & Glen, H. (1995). ZULULAND AHOY!. *Metamorphosis, 6*(1), 5-7.

Puttick, A., Bains, T., Böhm, M. & Müller, C.J. (2021). Pachliopta polyphontes. The IUCN Red List of Threatened Species 2021:

e.T121973688A122602271. https://dx.doi.org/10.2305/IUCN.UK.2021-2.RLTS.T121973688A122602271.en.

Puttick, A. & Böhm, M. (2018). Battus ingenuus. The IUCN Red List of Threatened Species 2018: e.T160495A836455. https://dx.doi.org/10.2305/IUCN.UK.2018-2.RLTS.T160495A836455.en.

Puttick, A. & Böhm, M. (2018). Battus laodamas. The IUCN Red List of Threatened Species 2018: e.T110516396A122602096. https://dx.doi.org/10.2305/IUCN.UK.2018-2.RLTS.T110516396A122602096.en.

Puttick, A. & Freitas, A.V.L. (2018). Battus lycidas. The IUCN Red List of Threatened Species 2018: e.T110519867A110520091. https://dx.doi.org/10.2305/IUCN.UK.2018-2.RLTS.T110519867A110520091.en.

Puttick, A. & Hall, P. (2018). Battus eracon. The IUCN Red List of Threatened Species 2018: e.T110515696A110515726. https://dx.doi.org/10.2305/IUCN.UK.2018-2.RLTS.T110515696A110515726.en.

Puttick, A., Hall, P. & Shuey, J. (2020). Papilio aristodemus. The IUCN Red List of Threatened Species 2020: e.T62156A110591422. https://dx.doi.org/10.2305/IUCN.UK.2020-2.RLTS.T62156A110591422.en.

Puttick, A., Hall, P. & Shuey, J. (2020). Papilio cresphontes. The IUCN Red List of Threatened Species 2020: e.T160091A813920. https://dx.doi.org/10.2305/IUCN.UK.2020-2.RLTS.T160091A813920.en.

Puttick, A., Hall, P. & Shuey, J. (2020). Papilio ornythion. The IUCN Red List of Threatened Species 2020: e.T160604A842619. https://dx.doi.org/10.2305/IUCN.UK.2020-2.RLTS.T160604A842619.en.

Puttick, A., Hall, P. & Shuey, J. (2020). Papilio rumiko. The IUCN Red List of Threatened Species 2020: e.T122548670A122603316. https://dx.doi.org/10.2305/IUCN.UK.2020-2.RLTS.T122548670A122603316.en.

Puttick, A. & Leon-Cortes, J. (2018). Papilio esperanza. The IUCN Red List of Threatened Species 2018: e.T15988A110606677. https://dx.doi.org/10.2305/IUCN.UK.2018-2.RLTS.T15988A110606677.en.

Puttick, A., Leon-Cortes, J. & Legal, L. (2018). Baronia brevicornis. The IUCN Red List of Threatened Species 2018: e.T2594A119581233. https://dx.doi.org/10.2305/IUCN.UK.2018-2.RLTS.T2594A119581233.en.

Puttick, A. & Nuñez, R. (2018). Battus devilliers. The IUCN Red List of Threatened Species 2018: e.T110515634A110515664. https://dx.doi.org/10.2305/IUCN.UK.2018-2.RLTS.T110515634A110515664.en.

Puttick, A. & Nuñez, R. (2018). Heraclides andraemon. The IUCN Red List of Threatened Species 2018: e.T110590900A110590920. https://dx.doi.org/10.2305/IUCN.UK.2018-2.RLTS.T110590900A110590920.en.

Puttick, A. & Nuñez, R. (2018). Heraclides caiguanabus. The IUCN Red List of Threatened Species 2018: e.T15984A110606481. https://dx.doi.org/10.2305/IUCN.UK.2018-2.RLTS.T15984A110606481.en.

Puttick, A. & Nuñez, R. (2018). Protographium celadon. The IUCN Red List of Threatened Species 2018: e.T110717766A110717796. https://dx.doi.org/10.2305/IUCN.UK.2018-2.RLTS.T110717766A110717796.en.

Puttick, A. & Nuñez, R. (2020). Heraclides aristor. The IUCN Red List of Threatened Species 2020: e.T15982A798819. https://dx.doi.org/10.2305/IUCN.UK.2020-2.RLTS.T15982A798819.en.

Puttick, A. & Nuñez, R. (2020). Papilio machaonides. The IUCN Red List of Threatened Species 2020: e.T110607935A110607939. https://dx.doi.org/10.2305/IUCN.UK.2020-2.RLTS.T110607935A110607939.en.

Puttick, A. & Nuñez, R. (2020). Papilio oxynius. The IUCN Red List of Threatened Species 2020: e.T110713738A110714557. https://dx.doi.org/10.2305/IUCN.UK.2020-2.RLTS.T110713738A110714557.en.

Puttick, A. & Nuñez, R. (2020). Papilio pelaus. The IUCN Red List of Threatened Species 2020: e.T110610772A110610776. https://dx.doi.org/10.2305/IUCN.UK.2020-2.RLTS.T110610772A110610776.en.

Puttick, A., Nunez-Bustos, E. & Freitas, A.V.L. (2018). Battus belus. The IUCN Red List of Threatened Species 2018: e.T159775A795945. https://dx.doi.org/10.2305/IUCN.UK.2018-2.RLTS.T159775A795945.en.

Puttick, A., Nunez-Bustos, E. & Freitas, A.V.L. (2018). Battus crassus. The IUCN Red List of Threatened Species 2018: e.T161079A871762. https://dx.doi.org/10.2305/IUCN.UK.2018-2.RLTS.T161079A871762.en.

Puttick, A., Rosa, A., Mega, N., Marini-Filho, O. & Freitas, A.V.L. (2020). Papilio astyalus. The IUCN Red List of Threatened Species 2020: e.T122524195A122602841. https://dx.doi.org/10.2305/IUCN.UK.2020-2.RLTS.T122524195A122602841.en.

Puttick, A., Walker, A. & Hall, P. (2021). Papilio polyxenes. The IUCN Red List of Threatened Species 2021: e.T110613568A110613582. https://dx.doi.org/10.2305/IUCN.UK.2021-1.RLTS.T110613568A110613582.en.

Puttick, A., Walker, A. & Shuey, J. (2021). Papilio multicaudata. The IUCN Red List of Threatened Species 2021: e.T110608304A110608309. https://dx.doi.org/10.2305/IUCN.UK.2021-1.RLTS.T110608304A110608309.en.

Racheli, T. (1979). New subspecies of *Papilio* and *Graphium* from the Solomon Islands, with Observations on *Graphium codrus* (Lepidoptera, Papilionidae). *Zoologische Mededelingen, 54*(15), 237-240.

Racheli, T. (1980). A list of the Papilionidae (Lepidoptera) of the Solomon Islands, with notes on their geographical distribution. *The Australian Entomologist, 7*(4), 45.

Racheli, T. (1995). An annotated check-list of Venezuelan Papilionidae (Lepidoptera). *Lambillionea, 95*(1), 70-80.

Racheli, T. (2006). *Butterflies of the world: The genus Parides. Supplement 13*. Keltern Goecke & Evers. 116 pp.

Racheli, T. & Biondi, M. (1989). Biogeographical observations on the Philippine Papilionoidea (Lepidoptera). *Italian Journal of Zoology, 56*(4), 333-347.

Racheli, T. & Cotton, A. M. (2009). *Guide to the butterflies of the Palearctic region Papilionidae part 1. Subfamily Papilioninae, tribes Leptocircini, Teinopalpini*. Milano: Omnes Artes. 70 pp.

Racheli, T. & Cotton, A. M. (2010). *Guide to the butterflies of the Palearctic region Papilionidae part 2. Subfamily Papilioninae, tribes Troidini.* Milano: Omnes Artes. 86 pp.

Racheli, T. & Pariset, L. (1993). An annotated check-list of Ecuadorian Papilionidae. *Atalanta 23*(3/4), 423-447.

Rafi, M. A., Khan, M. R. & Irshad, M. (2000). *Papilionid (swallowtails) butterflies of Pakistan*. Islamabad: Pakistan Agricultural Research Center. 33 pp.

Rassami, W., Koolkalya, S., Chaiyakul, K., & Sawarit, S. (2017). Species Diversity of Insect Pollinators in the Area of Plant Genetics Conservation Project under the Royal Initiation of Her Royal Highness Princess Maha Chakri Sirindhorn (RSPG) at the Rambhai Barni Rajabhat University, Chanthaburi Province, Thailand. *International Journal of Agricultural Technology, 13*(7.1), 1259-1267.

Rose, K. (2002). Zur Verbreitung und subspezifischen Gliederung von Parnassius cephalus Grum-Grshimailo, 1891 in China (Lepidoptera: Papilionidae). *Nachrichten des Entomologischen Vereins Apollo, Frankfurt am Main, NF 23*(1/2), 33-42.

Rumbucher, K. (1999a). *Butterflies of the World, Part 6: Papilionidae III:* Troides *I*. Keltern: Goecke & Evers. 34 pp.

Rumbucher, K. (1999b). *Butterflies of the World, Part 7: Papilionidae IV:* Troides *II*. Keltern: Goecke & Evers. 43 pp.

Rumbucher, K., Schäffler, O., Bauer, E. & Frankenbach, T. (2004) *Butterflies of the World, Part 19: Papilionidae X:* Troides *III*. Goecke & Evers, Keltern. 25 pp.

Rumbucher, K., Schäffler, O., Bauer, E. & Frankenbach, T. (2005) *Butterflies of the World, Part 21: Papilionidae XI:* Troides *IV*. Goecke & Evers, Keltern. 62 pp.

Rushbrooke, M., Jangid, A.K., Alwis, C., Barve, V., Chowdhury, S., Irungbam, J.S., Jayasinghe, H.D., Kehimkar, I., Khanal, B., Kunte, K., Lo, P., Moonen, J., Rajapakshe, S.S., Shrestha, B.R. & Tiple, A.D. (2020). Pachliopta aristolochiae (amended version of 2019 assessment). The IUCN Red List of Threatened Species 2020: e.T121971639A170544412. https://dx.doi.org/10.2305/IUCN.UK.2020-2.RLTS.T121971639A170544412.en.

Rushbrooke, M., Jangid, A.K., Kehimkar, I., Kunte, K. & Moonen, J. (2020). Losaria rhodifer (amended version of 2019 assessment). The IUCN Red List of Threatened Species 2020: e.T121974900A170538804. https://dx.doi.org/10.2305/IUCN.UK.2020-2.RLTS.T121974900A170538804.en.

Rushbrooke, M., Lo, P. & Moonen, J. (2020). Byasa impediens (amended version of 2019 assessment). The IUCN Red List of Threatened Species 2020: e.T121972089A176106484. https://dx.doi.org/10.2305/IUCN.UK.2020-3.RLTS.T121972089A176106484.en.

Rushbrooke, M. & Moonen, J. (2019). Pachliopta adamas. The IUCN Red List of Threatened Species 2019: e.T121971496A122602106. https://dx.doi.org/10.2305/IUCN.UK.2019-3.RLTS.T121971496A122602106.en.

Rushbrooke, M. & Moonen, J. (2020). Losaria neptunus (amended version of 2019 assessment). The IUCN Red List of Threatened Species 2020: e.T121973550A176625734. https://dx.doi.org/10.2305/IUCN.UK.2020-3.RLTS.T121973550A176625734.en.

Rushbrooke, M., Moonen, J. & Racheli, T. (2019). Pachliopta antiphus. The IUCN Red List of Threatened Species 2019: e.T121971622A122602126. https://dx.doi.org/10.2305/IUCN.UK.2019-3.RLTS.T121971622A122602126.en.

Rushbrooke, M., Moonen, J. & Racheli, T. (2019). Pachliopta kotzebuea. The IUCN Red List of Threatened Species 2019: e.T121972097A122602181. https://dx.doi.org/10.2305/IUCN.UK.2019-3.RLTS.T121972097A122602181.en.

Rushbrooke, M., Moonen, J. & Racheli, T. (2019). Pachliopta phlegon. The IUCN Red List of Threatened Species 2019: e.T121973605A122602246. https://dx.doi.org/10.2305/IUCN.UK.2019-3.RLTS.T121973605A122602246.en.

Rushbrooke, M., Moonen, J. & Racheli, T. (2020). Atrophaneura atropos (amended version of 2019 assessment). The IUCN Red List of Threatened Species 2020: e.T2375A176835285. https://dx.doi.org/10.2305/IUCN.UK.2020-3.RLTS.T2375A176835285.en.

Rushbrooke, M., Moonen, J. & Peggie, D. (2020). Papilio diophantus. The IUCN Red List of Threatened Species 2020: e.T122527368A122602936. https://dx.doi.org/10.2305/IUCN.UK.2020-2.RLTS.T122527368A122602936.en.

Rushbrooke, M., Moonen, J. & Peggie, D. (2020). Papilio lampsacus. The IUCN Red List of Threatened Species 2020: e.T122540322A122603116. https://dx.doi.org/10.2305/IUCN.UK.2020-2.RLTS.T122540322A122603116.en.

Rushbrooke, M. & Racheli, T. (2020). Pachliopta leytensis (amended version of 2019 assessment). The IUCN Red List of Threatened Species 2020: e.T121972240A176835590. https://dx.doi.org/10.2305/IUCN.UK.2020-3.RLTS.T121972240A176835590.en.

Sáfián, S. Z., Collins, S. C., Kormos, B. & Siklósi, A. (2009). *African Butterfly Database version 1.0*. Available at http://www.abdb-africa.org .(Accessed 2017/07/10).

Sakai, S., Inaoka, S., Aoki, T., Yamaguchi, S. & Watanabe, Y. (2002). *The parnassiology. The Parnassius Butterflies, A Study in Evolution.* Tokyo: Kodansha. 288 pp.

Samraoui, B. (1998). Status and seasonal patterns of adult Rhopalocera (Lepidoptera) in north-eastern Algeria. *Nachrichten des Entomologischen Vereins Apollo, 19*(3/4), 285-298.

Samson, C. (1982). Two new subspecies of Papilio canopus (Lepidoptera: Papilionidae) from the Solomons and Vanuatu. *Pacific insects, 24*(3-4), 228-231.

Savela, M. (2002). *Ecology of Lepidoptera database on the Finnish University and Research Network (FUNET).* Available at: http://www. funet. fi/pub/sci/bio/life/intro. (Accessed 2017/07/10).

Schäffler, O. & Rumbucher, K. (2014) *Butterflies of the World, Part 41: Papilionidae XV:* Troides *V and* Trogonoptera. Keltern: Goecke & Evers. 72 pp.

Schäffler, O., Bauer, E. & Frankenbach, T. (2001). *Butterflies of the World, Part 12:* Ornithoptera. Keltern: Goecke & Evers. 40 pp.

Schaus, W. & Clements, W. G. (1893). *On a Collection of Sierra Leone Lepidoptera*. London: R. H. Porter. 46 pp.

Schlicht, D. W., Downey, J. C. & Nekola, J. C. (2007). *The butterflies of Iowa*. Iowa City: University of Iowa Press. 252 pp.

Scott, J. A. (1992). *The butterflies of North America: a natural history and field guide*. Palo Alto: Stanford University Press. 664 pp.

Seraphim, N., Barreto, M. A., Almeida, G. S. S., Esperanço, A. P., Monteiro, R. F., Souza, A. P., Freitas, A. V. L. & Silva-Brandão, K. L. (2016). Genetic diversity of Parides ascanius (Lepidoptera: Papilionidae: Troidini): implications for the conservation of Brazil's most iconic endangered invertebrate species. Conservation genetics, 17(3), 533-546.

Singh, I. J. & Chib, M. S. (2015). Checklist of Butterflies of Bhutan. *Journal of the Bhutan Ecological Society, 1*(2), 22-58.

Smetacek, P. (2011). On the Anomalous Altitudinal Distribution of West Himalayan Troidini and Papilionini (Papilionidae). *Journal of the Lepidopterists' Society, 65*(2), 126-132.

Smetacek, P. (2012). Butterflies (Lepidoptera: Papilionoidea and Hesperoidea) and other protected fauna of Jones Estate, a dying watershed in the Kumaon Himalaya, Uttarakhand, India. *Journal of Threatened Taxa, 4*(9), 2857-2874.

Smetacek, P. (2015). *The Papilionid Butterflies of the Indian Subcontinent (Concise Edition*). Bhimtal & New Delhi: Buttefly Research Centre & Indinov Publishing. 120 pp.

Smith, C. R. & Vane-Wright, R. I. (2001). A review of the afrotropical species of the genus *Graphium* (Lepidoptera: Rhopalocera: Papilionidae). *Bulletin of the Natural History Museum, 70*, 503-719.

Smith, C. R., Liseki, S. & Vane-Wrght, R. I. (2008). On the status of *Papilio sjoestedti* Aurivillius, 1908, the "Kilimanjaro swallowtail" (Lepidoptera: Papilionidae). *Journal of Natural History, 42*(19-20), 1349-1359.

Song, W., Fang-yin, B., Bai-mao, M., & Shi-chao, D. (2009). Vertical distribution and community diversity of butterflies in Yaoluoping National Nature Reserve, Anhui, China. *Yingyong Shengtai Xuebao, 20*(9), 2262-2270.

Spencer, L. A. & Simons, D. R. (2006). *Arkansas butterflies and moths*. Fayetteville: University of Arkansas Press. 300 pp.

Sperling, F. A. H. & Harrison, R. G. (1994). Mitochondrial DNA variation within and between species of the *Papilio machaon* group of swallowtail butterflies. *Evolution, 48*(2), 408-422.

Takara, T. (1956). Provisional list of butterflies in the Ryukyu Islands. *Science Bulletin of the Agriculture and Home Economics Division, Okinawa, 3*, 34-122.

Thakur, M. S., Mehta, H. S. & Mattu, V. K. (2002). Butterflies of Kalatop-Khajjiar Wildlife Sanctuary, Himachal Pradesh. *Zoos' Print Journal, 17*(10), 909-910.

Thompson, M. J. & Timmermans, M. J. T. N. (2014). Characterising the phenotypic diversity of *Papilio dardanus* wing patterns using an extensive museum collection. *PLoS ONE, 9*(5), e96815.

Timberlake, J., Bayliss, J., Alves, T., Baena, S., Francisco, J., Harris, T. & da Sousa, C. (2007). *The biodiversity and conservation of Mount Chiperone, Mozambique*. Report produced under the Darwin Initiative Award 15/036: Monitoring and Managing Biodiversity Loss in South-East Africa's Montane Ecosystems. London: Royal Botanic Gardens, Kew. 33 pp.

Tolman, T. & Lewigton, R. (2004) Collins Field Guide – Butterflies of Britain and Europe. New York: HarperCollins Publishers. 320 pp.

Touroult, J., Deknuydt, F., Poirier, E., Rome, D., Ravat, P. & Neild, A. (2014). *Heraclides androgeus*: un nouveau Papilionidae pour la faune de Martinique et confirmation de sa présence à Sainte-Lucie (Lepidoptera Papilionoidea). *L'Entomologiste, 70*(2), 73-77.

Tshikolovets, V. V. (1998). *The Butterflies of Turkmenistan*. Kyiv: Nacional Na Akademija Nank Ukrainy. 237 pp.

Tshikolovets, V. V. (2011). *Butterflies of Europe & the Mediterranean area*. Kyiv & Brno: Nacional Na Akademija Nank Ukrainy. 544 p.

Tshikolovets, V. V. & Nekrutenko, Y. P. (2000). *The butterflies of Uzbekistan*. Kyiv & Brno: Nacional Na Akademija Nank Ukrainy. 399 pp.

Tshikolovets, V. V., Bidzilya, O. V. & Golovushkin, M. I. (2002). *Butterflies of Transbaikal, Siberia*. Kyiv & Brno: Nacional Na Akademija Nank Ukrainy. 320 pp.

Tshikolovets, V. V., Naderi, A. & Eckweiler, W. (2014). *The butterflies of Iran and Iraq*. Kyiv & Brno: Tshikolovets Publications. 367 pp.

Tsukada, E. & Nishiyama, Y. (1982). *Butterflies of the South East Asian Islands. Vol 1: Papilionidae*. Tokyo: Plapac Co. 457 pp.

Tyler, H. A., Brown, K. S. & Wilson, K. H. (1994). *Swallowtail butterflies of the Americas*. Gainesville: Scientific Publishers. 376 pp

Uemura, Y. (2016). A synonymic List of Butterflies of Bhutan. Part I. Papilionidae. *Bullefin of the Toyosato Museum of Entomology, 25*, 1-10.

UNEP-WCMC. (2012) *Review of butterflies from Asia and Oceania subject to long-standing positive opinions*. UNEP-WCMC, Cambridge. 100 pp.

van Swaay, C., Cuttelod, A., Collins, S., Maes, D., Munguira, M. L., Šašić, M., Settele, J., Verovnik, R., Verstrael, T., Warren, M., Wiemers, M. & Wynhoff, I. (2010). European red list of butterflies. Luxembourg: Publications Office of the European Union. 47 pp.

van Swaay, C., Wynhoff, I., Wiemers, M., Katbeh-Bader, A., Power, A., Benyamini, D., Tzirkalli, E., Balletto, E., Monteiro, E., Karaçetin, E., Franeta, F., Pe'er, G., Welch, H., Thompson, K., Pamperis, L., Dapporto, L., Šašić, M., López Munguira, M., Micevski, N., Dupont, P., Garcia-Pereira, P., Moulai, R., Caruana, R., Verovnik, R., Bonelli, S. & Beshkov, S. (2015). Papilio hospiton. The IUCN Red List of Threatened Species 2015: e.T15993A64822977. https://dx.doi.org/10.2305/IUCN.UK.2015-2.RLTS.T15993A64822977.en.

Vane-Wright, R. I. & de Jong, R. (2003). *The butterflies of Sulawesi: annotated checklist for a critical island fauna*. Leiden: Nationaal Natuurhistorisch Museum. 267 pp.

Vane-Wright, R. I. & Liseki, S. (2008). The type material, taxonomy and conservation of Horniman's Swallowtail, *Papilio hornimani* (Lepidoptera: Papilionidae). *Journal of Natural History, 42*(19-20), 1333-1348.

Vargas-Fernández, I., Luis-Martínez, A. & Llorente-Bousquets, J. (2013). A new subspecies of *Heraclides androgeus* (Lepidoptera: Papilionidae) and its biogeographical aspects. *Revista de Biología Tropical, 61*(2), 711-733.

Varshney, R. K. (1993). Index Rhopalocera Indica Part III. Genera of butterflies from India and neighbouring countries (Lepidoptera:(A) Papilionidae, Pieridae and Danaidae). *Oriental Insects, 27*(1), 347-372.

Varshney, R. K. & Smetacek, P. (2015). *A Synoptic Catalogue of the Butterflies of India*. New Delhi: Bhimtal and Indinov Publishing. 261 pp.

Walker, D. H., Pittaway, A. R. & Walker, A. J. (1987). *Insects of eastern Arabia.* London: Macmillan Education. 191 pp.

Wangdi, K. (2012). *Field Guide for Swallowtails of Bhutan*. Bumthang: Ugyen Wangchuk Institute for Conservation and Environment. 77 pp.

Wautelet, F., Dulfost, J. & Cigar, J. (2005). Albertine Rift-Biodiversity data. Available at http://projects.bebif.be/enbi/albertinerift/common/ (Accessed 2017/07/10)

Wei, Z. & Yang, Y. (2012) Species diversity of butterflies in Changbai Mountain in China. *Acta Ecologica Sinica, 32*(6), 279-284.

Weiss, J. C. (1999). *The Parnassiinae of the world, part 3*. Canterbury: Hillside Books. 99 pp.

Williams, J. G. (1969). *A field guide to the butterflies of Africa*. London: Collins. 238 pp.

Williams, M. C. (2004). *Butterflies and Skippers of the Afrotropical Region (Papilionoidea and Hesperioidea) an Encyclopedia.* Availabe at http://atbutterflies.com/. (Accessed 2017/07/10)

Willis, C. K & Woodhall, S. E (2010). *Butterflies of South Africa's National Botanical Gardens an illustrated*. Pretoria: South African National Biodiversity Institute. 232 pp.

Wilson, C. E. (1953). Butterflies of the southern Sudan. *Sudan Notes and Records, 34*, 73-103.

Wilson, J. J., Karen-Chia, H. M., Sing, K. W. & Sofian-Azirun, M. (2014). Towards resolving the identities of the Graphium butterflies (Lepidoptera: Papilionidae) of Peninsular Malaysia. *Journal of Asia-Pacific Entomology, 17*(3), 333-338.

Wiltshire, E. P. (1945). Studies in the geography of Lepidoptera – II. Swallowtails in desertic SW Asia. *Physiological Entomology, 20*(1-3), 16-25.

Woodhall, S. (2005). *Field guide to butterflies of South Africa*. Cape Town: Cape Town Struik. 440 pp.

Xu, Z. B., Wang, Y. Y., Condamine, F. L., Cotton, A. M., & Hu, S. J. (2020). Are the yellow and red marked club-tail Losaria coon the same species?. *Insects*, *11*(6), 392.

Yakovlev, R. V. (2012). Checklist of Butterflies (Papilionoidea) of the Mongolian Altai Mountains, including descriptions of new taxa. *Nota lepidopterological, 35*(1), 51-96.

Yong, D. L. Lohman, D. J. Weei Gan, C. Qie, L. & Lim, S. L. (2012) Tropical butterfly communities on land-bridge islands in peninsular Malaysia. *Raffles Bulletin of Zoology* Supplement, *25*, 161-172.

Yoshino, K. (2001). Notes of *Chilasa agestor* and *Pazala hoenei*, stat. nov. (Lepidoptera, Papilionidae) from South China. *Transactions of the Lepidopterological Society of Japan, 52*, 136-138

Zeng, J. P.; Zhou, S. Y.; Li, C. C.; Wu, J. S. & Qin, K. (2005). The pupa of *Teinopalpus aureus guangxiensis* and discovery of its host plant. *Kunchong Zhishi, 42*(1), 71-73

Zhang, W., Westerman, E., Nitzany, E., Palmer, S. & Kronforst, M. R. (2017). Tracing the origin and evolution of supergene mimicry in butterflies. *Nature communications, 8*(1), 1269.

Appendix S3

Table S3.1 Observed and simulated BSI values for Papilionidae. p: probability of the proportion of observed species being higher (bold), lower (italics) or non-significantly different (plain) to simulated proportions in Papilionidae.

**BSI**	**% Observed**	**Monte Carlo Analysis**			
		**Mean %**	**Std. dev.**	**Range**	**p**
**1**	54.50	38.00	1.60	32.00-45.00	**<0.001**
**2**	31.50	41.00	2.10	33.00-49.00	*<0.001*
**3**	9.95	17.00	1.40	11.00-24.00	*<0.001*
**4**	2.53	3.50	0.72	1.30-7.00	0.086
**5**	0.68	0.40	0.27	0.00-1.50	0.186
**6**	0.34	0.03	0.07	0.00-0.57	**0.009**
**7**	0.34	0.00	0.01	0.00-0.20	**<0.001**
**8**	0.17	0.00	0.00	0.00-0.00	**<0.001**
**9**	0.00	0.00	0.00	0.00-0.00	1.000
**10**	0.00	0.00	0.00	0.00-0.00	1.000

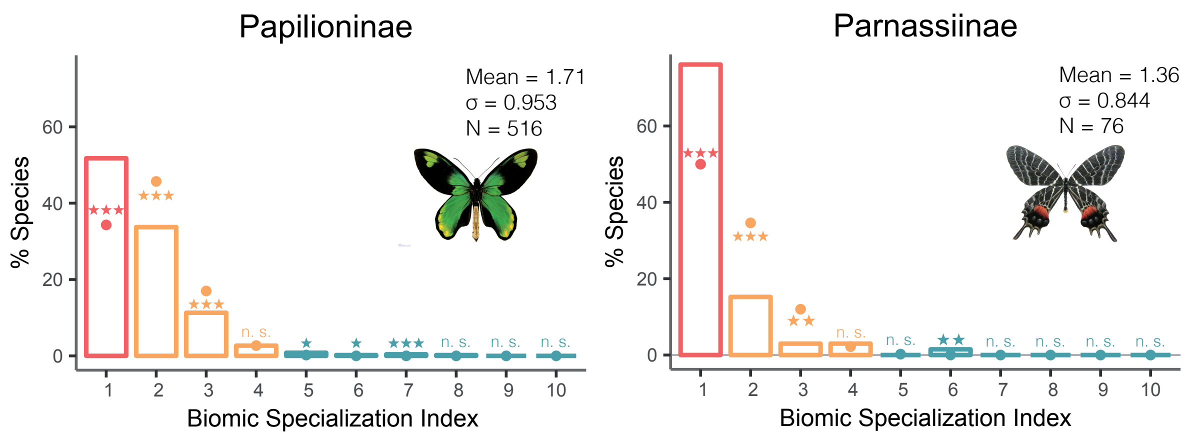

Figure S3.1. Frequency distribution of Biomic Specialization Index (BSI) in Papilionidae subfamilies: Papilioninae and Parnassiinae. Colours indicate the biomic specialization degree: red = specialist species; yellow = semi-generalist species; blue = extreme generalist species. Dots indicate the expected values by chance. *** = p<0,001; ** = 0,01>p>0,001; * = 0,05>p>0,01; n.s = not significant. Symbols above or below the dots indicate whether results are significantly higher (above) or lower (bellow) than expected by chance.

Table S3.2 Observed and simulated BSI values for Papilionidae subfamilies: Papilioninae and Parnassiinae. p: probability of observed species being higher (bold), lower (italic) or non-significantly different (plain) to simulated proportions.

**Papilioninae**						**Parnassiinae**				
**BSI**	**% Observed**	**Monte Carlo Analysis**				**% Observed**	**Monte Carlo Analysis**			
		**Mean %**	**Std. dev.**	**Range**	**p**		**Mean %**	**Std. dev.**	**Range**	**p**
**1**	51.00	36.00	1.60	28.00-42.00	**<0.001**	77.60	51.00	4.80	29.00-67.00	**<0.001**
**2**	33.90	44.00	2.20	37.00-54.00	*<0.001*	15.80	35.00	5.90	16.00-62.00	*<0.001*
**3**	11.00	17.00	1.40	11.00-23.00	*<0.001*	2.63	12.00	3.50	0.00-29.00	*0.001*
**4**	2.652	2.70	0.66	0.63-5.40	0.702	2.63	2.00	1.70	0.00-12.00	0.550
**5**	0.78	0.22	0.20	0.00-1.50	**0.014**	0.00	0.19	0.55	0.00-5.10	0.888
**6**	0.19	0.01	0.04	0.00-0.43	**0.004**	1.32	0.01	0.12	0.00-1.80	**0.006**
**7**	0.39	0.00	0.01	0.00-0.21	**<0.001**	0.00	0.00	0.00	0.00-0.00	1.000
**8**	0.19	0.00	0.00	0.00-0.00	1.000	0.00	0.00	0.00	0.00-0.00	1.000

**9**	0.00	0.00	0.00	0.00-0.00	1.000	0.00	0.00	0.00	0.00-0.00	1.000
**10**	0.00	0.00	0.00	0.00-0.00	1.000	0.00	0.00	0.00	0.00-0.00	1.000

Table S3.3. Observed and simulated distribution of biome specialist (BSI=1) Papilionidae species across different biomes. p: probability of the proportion of observed species being higher (bold), lower (italics) or non-significantly different (plain) to simulated proportions in Papilionidae among biomes.

**Biome**	**Papilionidae**			**Monte Carlo Analysis**			
	**Sp**	**Sp (BSI=1)**	**%**	**Mean %**	**Std. dev**	**Range**	**p**
**E.R**	331	143	43.20	24.70	1.90	16.30-32.30	**<0.001**
**T.W**	330	101	30.61	24.60	1.90	17.30-31.80	**<0.001**
**Sa**	38	0	0.00	11.70	5.20	0.00-34.20	*<0.001*
**T.D**	7	1	14.29	11.10	12.00	0.00-71.40	0.217
**S.W**	23	8	34.78	11.20	6.50	0.00-39.10	**<0.001**
**T.F**	117	15	12.82	13.60	3.00	2.56-27.40	0.556
**B.F**	52	6	11.54	12.00	4.40	0.00-30.80	0.777
**St**	58	40	68.97	12.10	4.30	0.00-32.80	**<0.001**
**Ta**	23	6	26.09	11.30	6.60	0.00-43.50	**0.013**
**Tu**	9	3	33.33	11.00	10.00	0.00-66.70	**0.010**

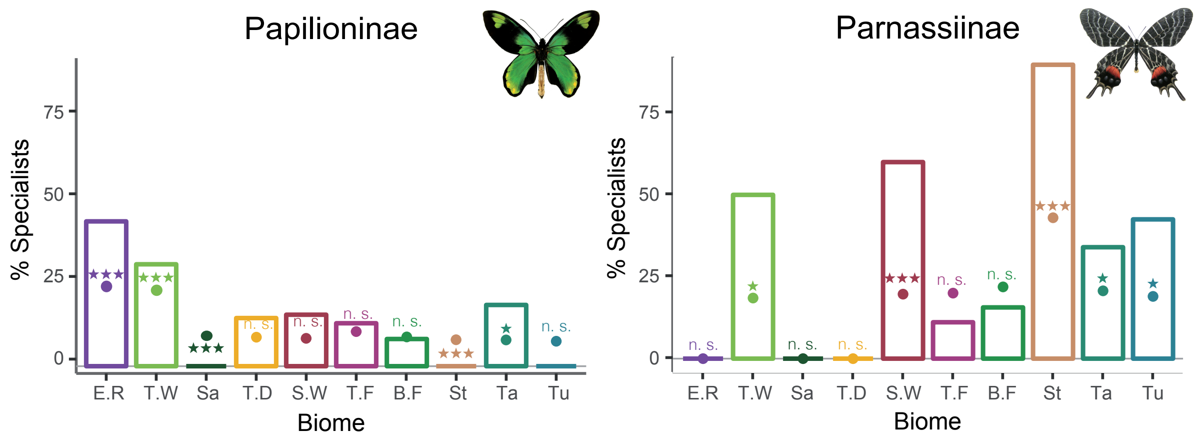

Figure S3.2. Observed and simulated distribution of biome specialist species (BSI=1) in Papilioninae and Parnassiinae species across different biomes. *** = p<0,001; ** = 0,01>p>0,001; * = 0,05>p>0,01; n.s = not significant. Symbols above or below the dots indicate whether results are significantly higher (above) or lower (bellow) than expected by chance. Colors for biomes as in Table 1 and Figure 6.

Biome	Papilioninae							Parnassiinae						
				Monte Carlo Analysis							Monte Carlo Analysis			
	Sp	Sp (BSI=1)	%	Mean %	Std. dev	Range	p	Sp	Sp (BSI=1)	%	Mean %	Std. dev	Range	p
E.R	331	143	43.20	23.00	1.80	16.90-29.30	**<0.001**	0	0	0.00	0.00	0.00	0.00-0.00	1.000
T.W	325	98	30.15	22.30	1.80	16.00-28.90	**<0.001**	4	2	50.00	19.10	20.00	0.00-100.00	**0.023**
Sa	38	0	0.00	8.95	4.50	0.00-28.90	*<0.001*	0	0	0.00	0.00	0.00	0.00-0.00	1.000
T.D	7	1	14.29	8.42	11.00	0.00-71.40	0.125	0	0	0.00	0.00	0.00	0.00-0.00	1.000
S.W	13	2	15.38	8.39	7.70	0.00-46.20	0.101	10	6	60.00	20.40	12.00	0.00-70.00	**<0.001**

**T.F**	108	14	12.96	10.50	2.80	0.93-22.20	0.172	9	1	11.11	20.00	13.00	0.00-77.80	0.142
**B.F**	36	3	8.33	8.86	4.70	0.00-36.10	0.666	16	3	18.75	22.70	9.80	0.00-62.50	0.317
**St**	13	0	0.00	8.41	7.70	0.00-46.20	*<0.001*	45	40	88.89	43.80	5.60	22.20-64.40	**<0.001**
**Ta**	11	2	18.18	8.48	8.20	0.00-45.50	**0.041**	12	4	33.33	21.30	11.00	0.00-66.70	**0.044**
**Tu**	2	0	0.00	7.93	19.00	0.00-100.00	0.180	7	3	42.86	15.00	15.00	0.00-85.70	**0.027**

Table S3.4. Observed and simulated distribution of biome specialist (BSI=1) Papilioninae and Parnassiinae species across different biomes. p: probability of the proportion of observed species being higher (bold), lower (italics) or non-significantly different (plain) to simulated proportions in Papilionidae subfamilies, Papilioninae and Parnassiinae, among biomes.

Table S3.5. DR (Jetz et al., 2012) diversification rate statistical values grouped by species's Biomic Specialization Index for all Papilionidae species present in the phylogeny.

BSI	DR Mean Value	DR Max Value	DR Min Value	DR Value Range	DR standard deviation
**1**	0.1560	0.6101	0.0189	0.5912	0.0908
**2**	0.1403	0.6101	0.0262	0.5839	0.1042
**3**	0.1324	0.3706	0.0259	0.3447	0.0821
**4**	0.1151	0.2674	0.0609	0.2065	0.0562
**5**	0.1340	0.2050	0.0502	0.1548	0.0709
**6**	0.0814	0.1126	0.0502	0.0624	0.0442
**7**	0.1323	0.1669	0.0977	0.0692	0.0346
**8**	0.1339	0.1339	0.1339	-	-

Table S3.6 Model outputs for Phylogenetic ANOVA analysis of the relationship between biomic specialization (BSI) and diversification rate, calculated under DR methodology.

DR					
	Df	Sum of Squares	Mean Square	F	p
Group	7	26.113	3.7304	4.1452	0.0002
Residual	191	171.887	0.8999		

Table S3.7. Summary of model support for null (CID), binary state speciation and extinction (BiSSE) and hidden state speciation and extinction (HiSSE) trait-dependent models. The coding indicates biomes specialist species as “1” and biome generalists, both moderate and extreme, as “0”. Best-fit model is highlighted in bold.

Model	NP	logL	AICc	∆AICc	lambda0A	lambda1A	lambda0B	lambda1B	mu0A	mu1A	mu0B	mu1B
CID model	5	-1.536.813	3083,781	40,025	0,1072		0,1070		<0,0001		<0,0001	
full BiSSE model	6	-1.529.103	3070,424	26,668	0,0570	0,1509	-	-	<0,0001	<0,0001	-	-
HiSSE model for state 0	10	-1514,16	3058,897	15,141	0,0591	0,1611	0,0480	-	<0,0001	<0,0001	<0,0001	-
**HiSSE model for state 1**	**10**	**-1511,59**	**3043,756**	**0**	**0,1867**	**0,0990**	**-**	**0,0941**	**<0,0001**	**<0,0001**	**0,0000**	**<0,0001**
full HiSSE model	16	-1.509.274	3061,994	18,238	0,0274	0,0860	0,1768	0,1655	0,0822	<0,0001	<0,0001	<0,0001

Model	q0A1A	q0A0B	q1A0A	q1A1B	q0B1A	q0B1B	q0B0A	q1B1A	q1B0B
CID model	2,5278								
full BiSSE model	0,0787	-	0,1184	-	-	-	-	-	-
HiSSE model for state 0	100,0000	0,1555	36,7148	-	-	-	3,37E-02	-	-
**HiSSE model for state 1**	**3,1265**	**-**	**4,7124**	**0,0077**	**-**	**-**	**-**	**0,0509**	**-**
full HiSSE model	0,0683	0,0010	0,2264	0,0000	0,0000	3,7900	<0,0001	0,1657	2,9203
